# Supplementary material for: Comprehensive Metagenomic Analysis of Veterinary Probiotics in Broiler Chickens
Source: Animals (Basel). 2024 Jun 29;14(13):1927. doi: 10.3390/ani14131927 (PMC11240415; doi:10.3390/ani14131927)
Supplement: Supplementary file 1 [file animals-14-01927-s001.zip › Supplementary materials.pdf]

Supplementary Table 1. Environmental parameters required during the rearing period of broiler chickens

**Day**	**Temperature °C**	**Humidity %**	**Length of lighting (hours)**
1	30	60-70%	24
2	30	60-70%	23
3	28	60-70%	23
4	28	60-70%	23
5	28	60-70%	23
6	27	60-70%	23
7	27	60-70%	18
8	27	60-70%	18
9	26	60-70%	18
10	26	60-70%	18
11	26	60-70%	18
12	25	60-70%	18
13	25	60-70%	18
14	25	60-70%	18
15	24	60-70%	18
16	24	60-70%	18
17	24	60-70%	18
18	23	60-70%	18
19	23	60-70%	18
20	23	60-70%	18
21	22	60-70%	18
22	22	60-70%	18
23	22	60-70%	18
24	21	60-70%	18
25	21	60-70%	18
26	21	60-70%	18
27	20	60-70%	18
28	20	60-70%	18
29	20	60-70%	18
30	20	60-70%	18
31	20	60-70%	18
32	20	60-70%	18
33	20	60-70%	18
34	20	60-70%	18
35	20	60-70%	18
36	20	60-70%	18
37	20	60-70%	18
38	20	60-70%	18
39	20	60-70%	18
40	20	60-70%	18
41	20	60-70%	18
42	20	60-70%	18

Supplementary Table 2. The main ingredients of broiler feed

Component	Starter	Grower	Finisher
	**0-14 Days**	**15-30 Days**	**31-42 Days**
Humidity	10.64%	11.47%	11.37%
Crude protein	20.96%	20.49%	19.01%
Crude fat	6.00%	6.32%	7.14%
Crude fiber	2.86%	3.17%	3.00%
Crude ash	6.07%	5.84%	5.66%
AMEn* poultry	12.03 MJ/kg	13.00 MJ/kg	13.33 MJ/kg
Lysine	1.25%	1.20%	1.11%
Methionine	0.54%	0.55%	0.53%
Calcium	1.05%	0.85%	0.84%
Phosphor	0.75%	0.66%	0.65%
Sodium	0.16%	0.17%	0.16%

* apparent metabolizable energy corrected to zero nitrogen retention

Supplementary Table 3. Statistics for groups and body mass at week 6, for all individuals with a body mass exceeding 2000 grams.

Groups	Mean different from Group I.	Standard Error	t-value	p-value
Group I.	2695.89	98.57	27.35	0.00
Group II.	137.41	135.87	1.01	0.32
Group III.	97.11	139.40	0.70	0.49
Group IV.	106.71	135.87	0.79	0.44
Group V.	22.41	135.87	0.16	0.87
Group VI.	83.44	139.40	0.60	0.55

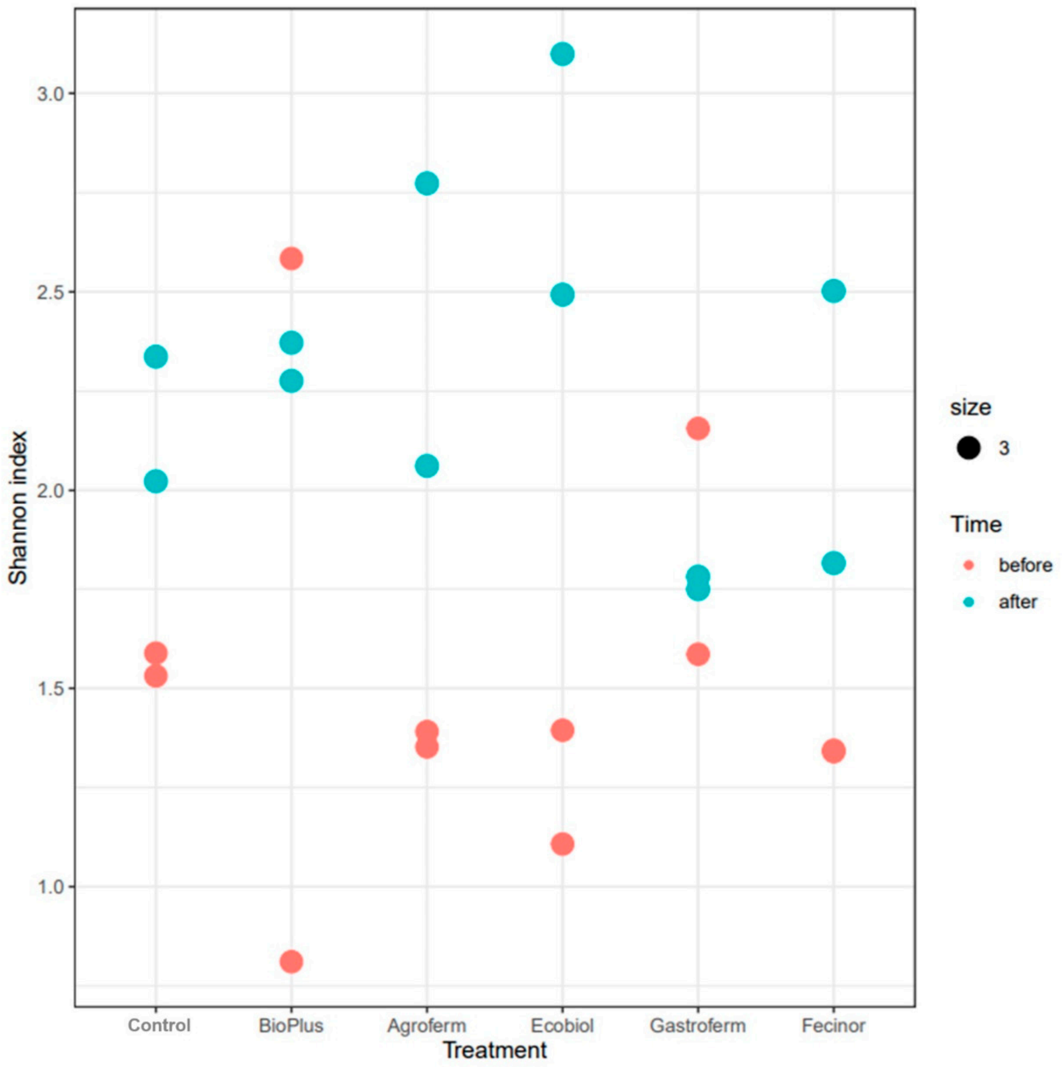

Supplementary Figure 1 Analysis of Shannon diversity distribution at the genus level by group revealed no significant differences between pre- and post-feeding samples when using a paired Mann-Whitney test within groups.

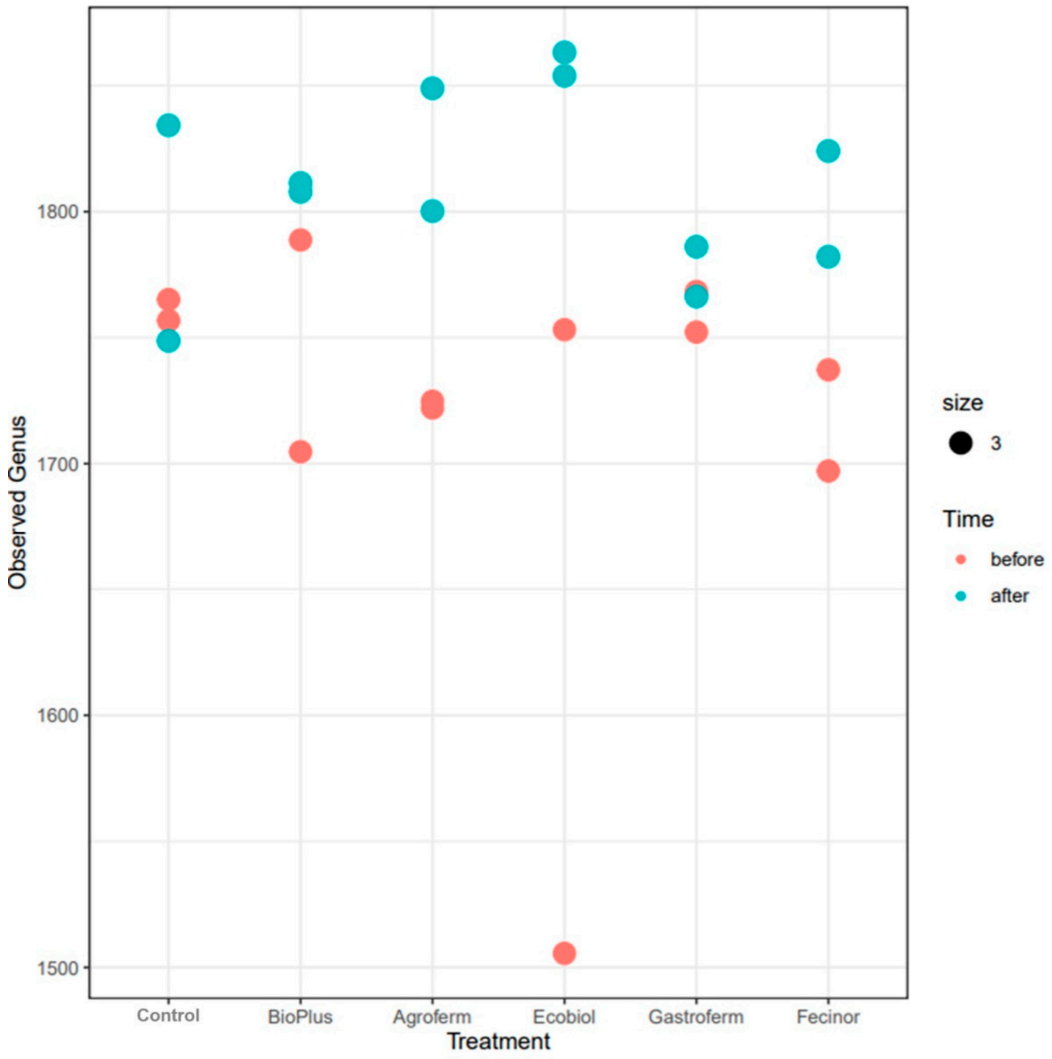

Supplementary Figure 2 Distribution of the number of genes observed across different sampling times showed no significant differences between pre- and post-feeding samples when analyzed using a paired Mann-Whitney test within groups.

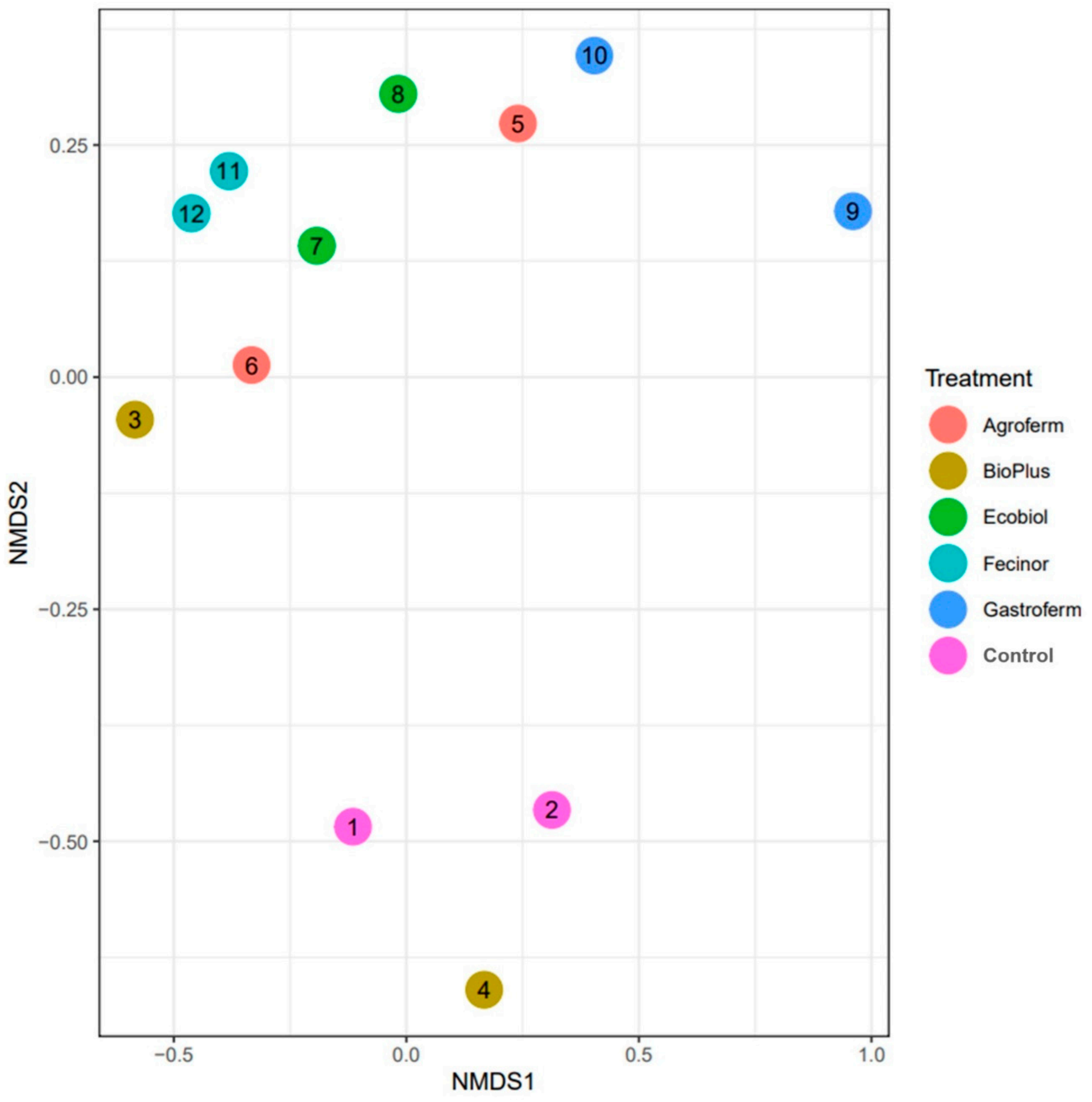

Supplementary Figure 3 Non-metric multidimensional scaling (NMDS) ordination based on Bray-Curtis distances between pre-feeding samples at the genus level.

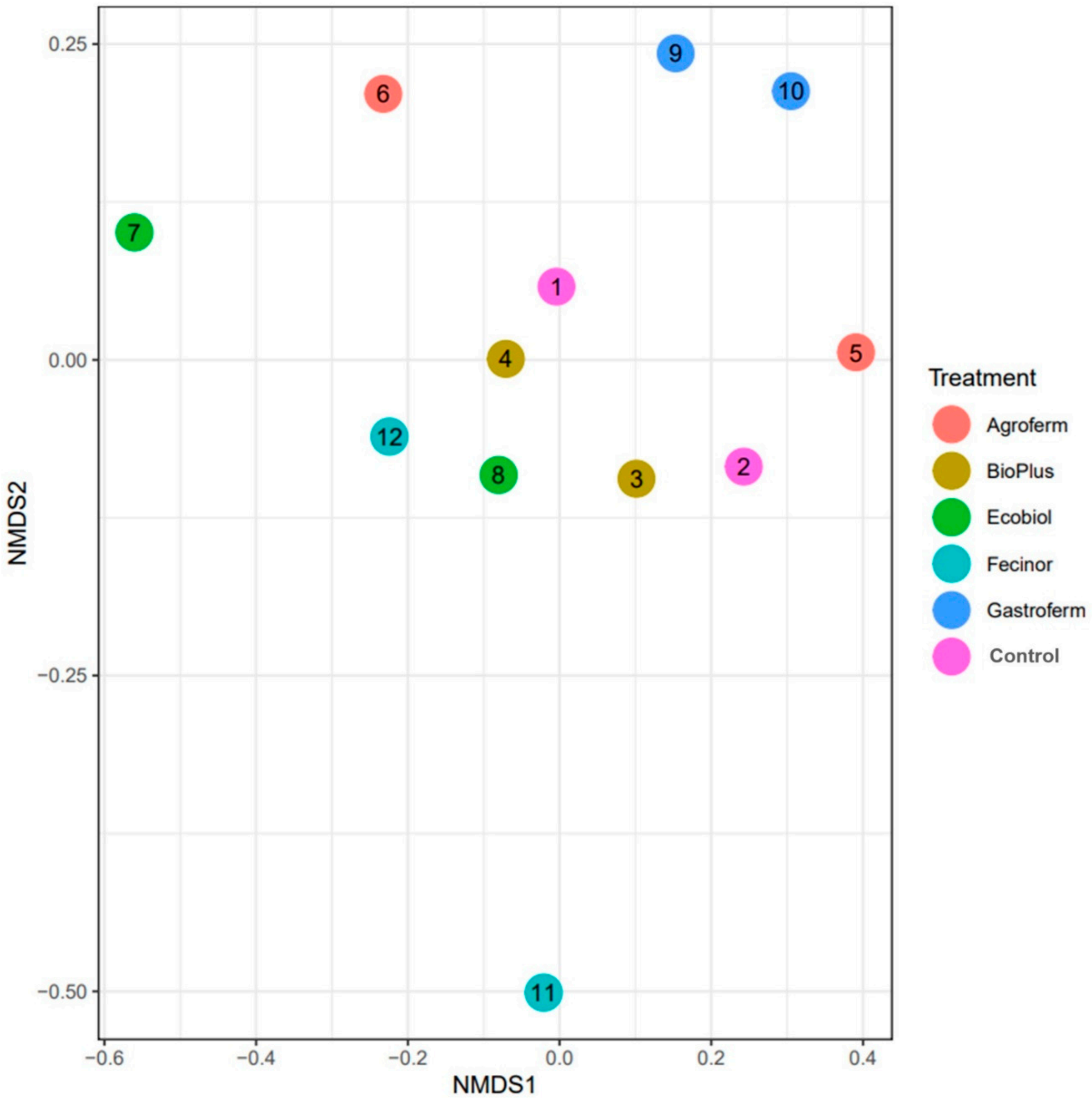

Supplementary Figure 4 Non-metric multidimensional scaling (NMDS) ordination of Bray-Curtis distances between post-feeding samples at the genus level.

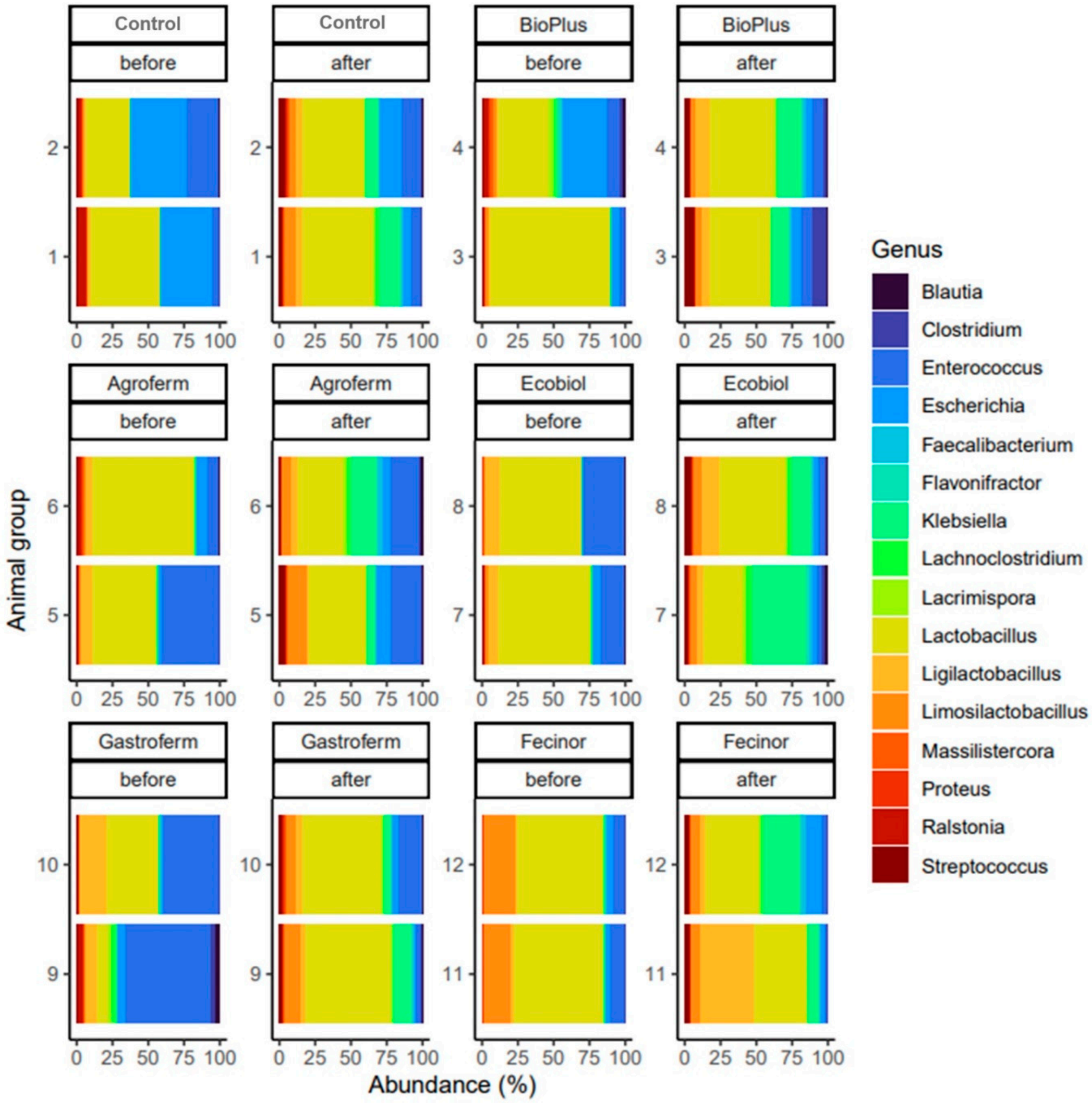

Supplementary Figure 5 Abundances of the core bacteriome per sample, for genes present in at least 10% of the samples at a minimum abundance of 1%.

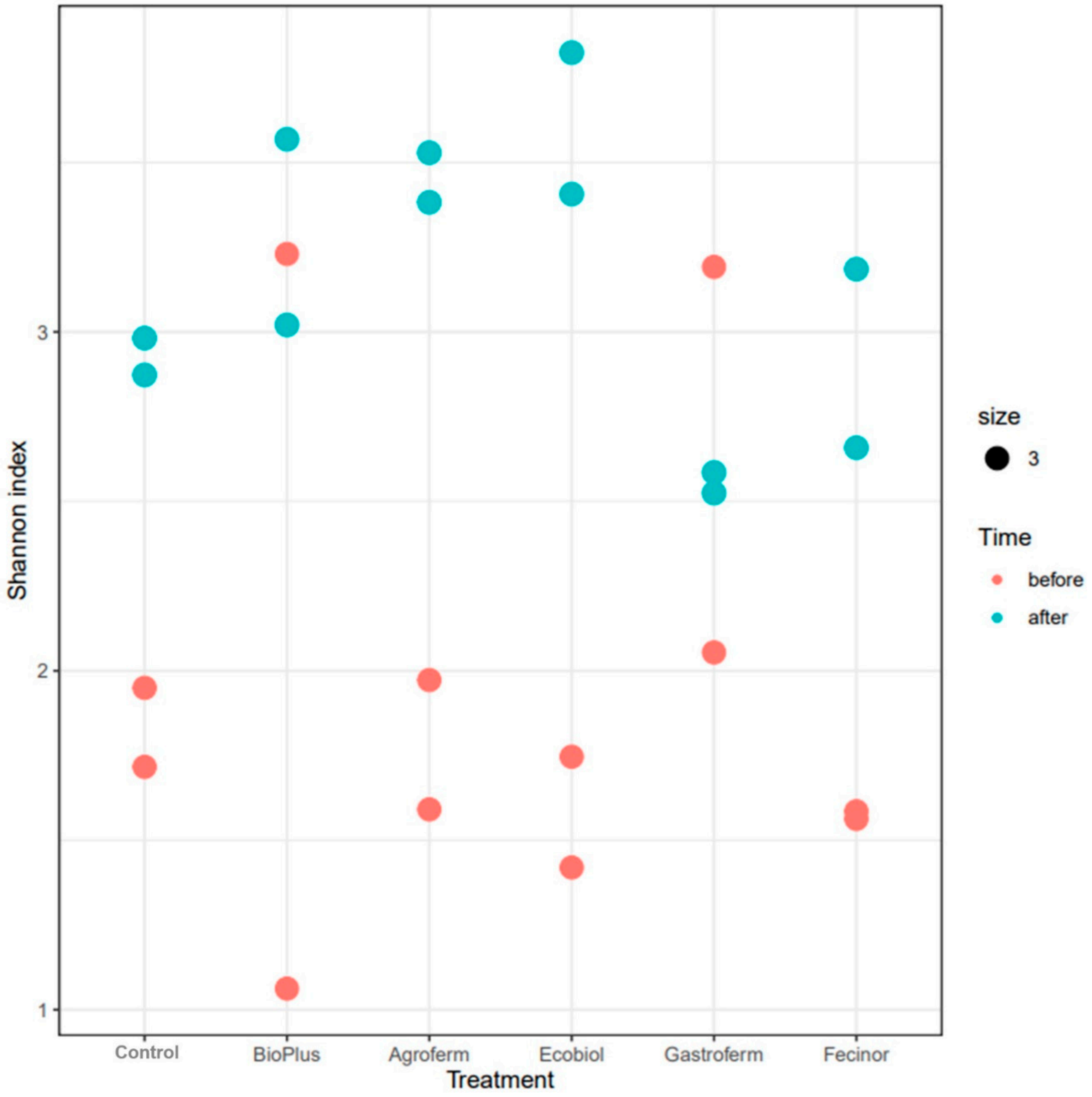

Supplementary Figure 6 Shannon's diversity distribution at the species level by group for probiotic-treated samples. No significant differences were detected by the paired Mann-Whitney test within groups.

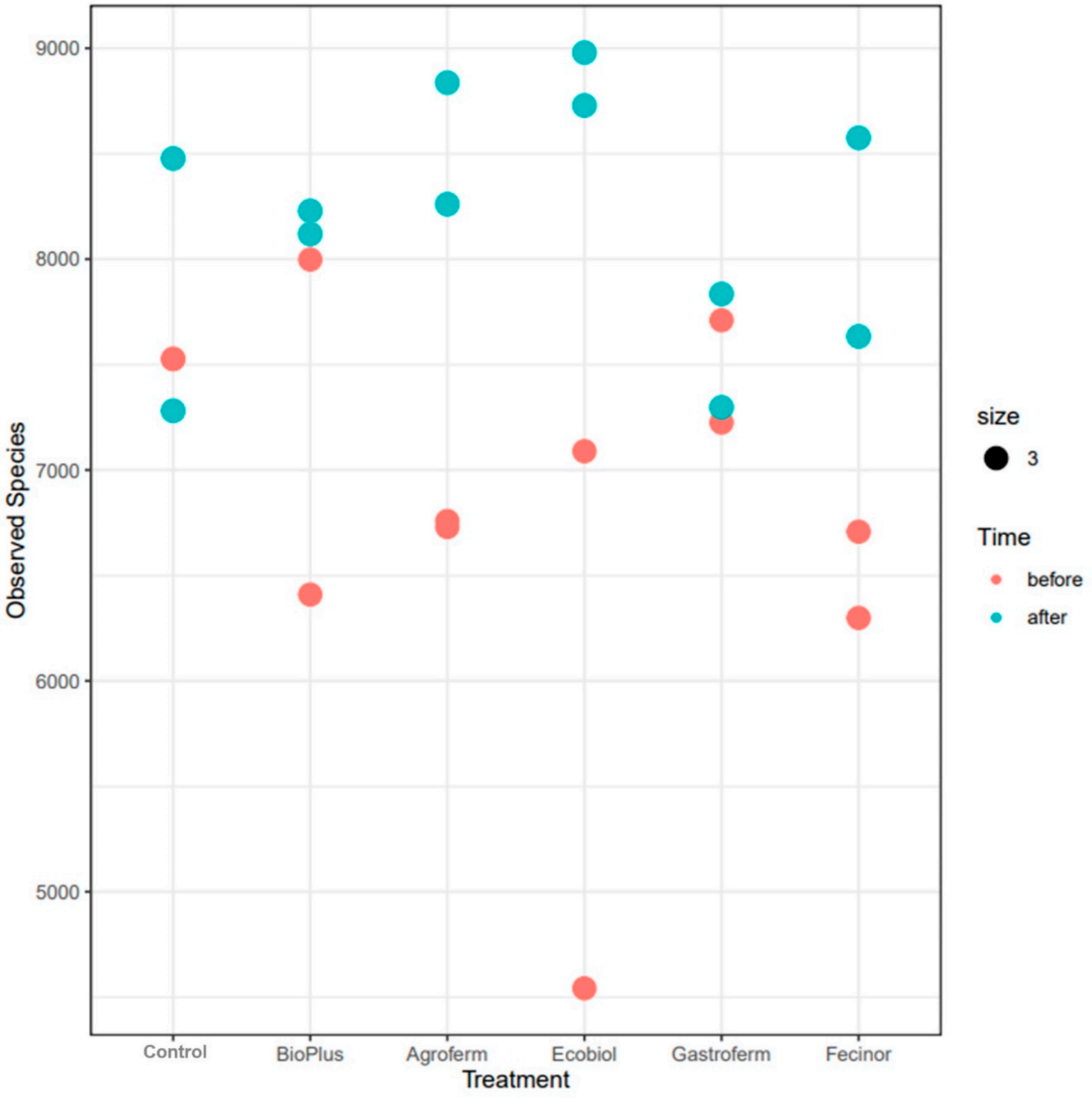

Supplementary Figure 7 Distribution of the number of species observed showed no significant differences when analyzed using the paired Mann-Whitney test within groups.

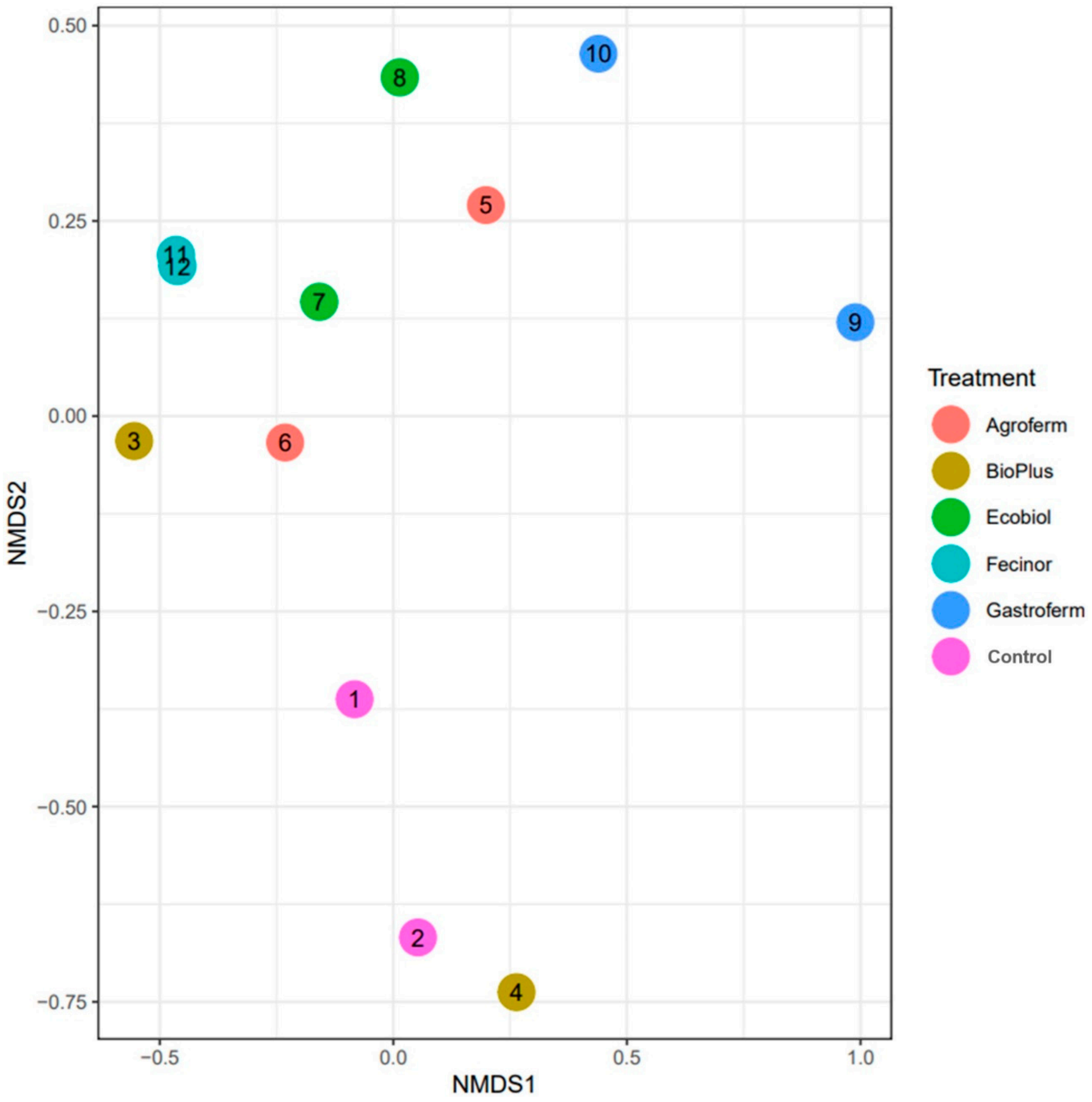

Supplementary Figure 8 Non-metric multidimensional scaling (NMDS) ordination based on Bray-Curtis distances among pre-feeding samples at the species level.

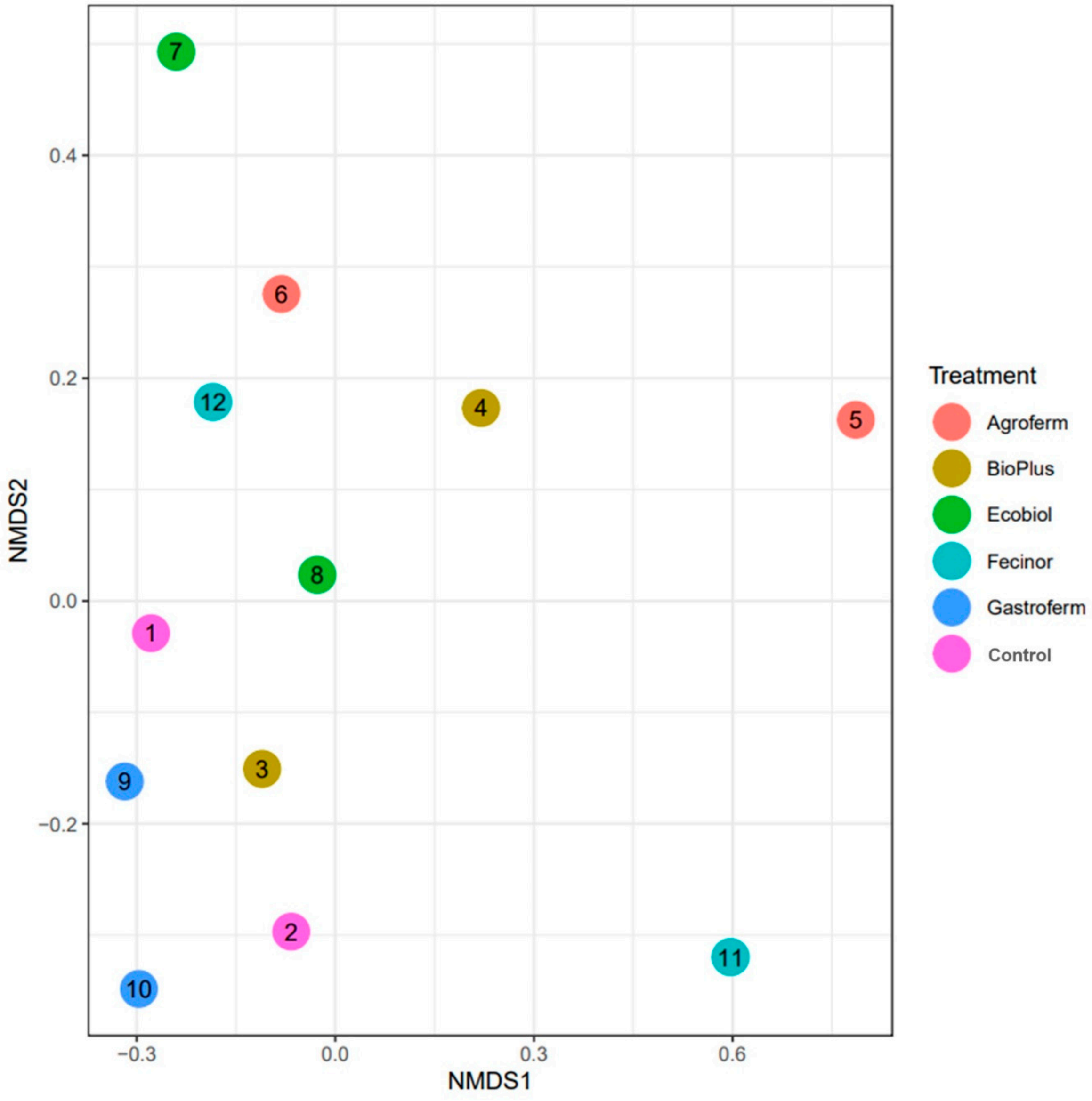

Supplementary Figure 9 Non-metric multidimensional scaling (NMDS) ordination based on Bray-Curtis distances among post-feeding samples at the species level.

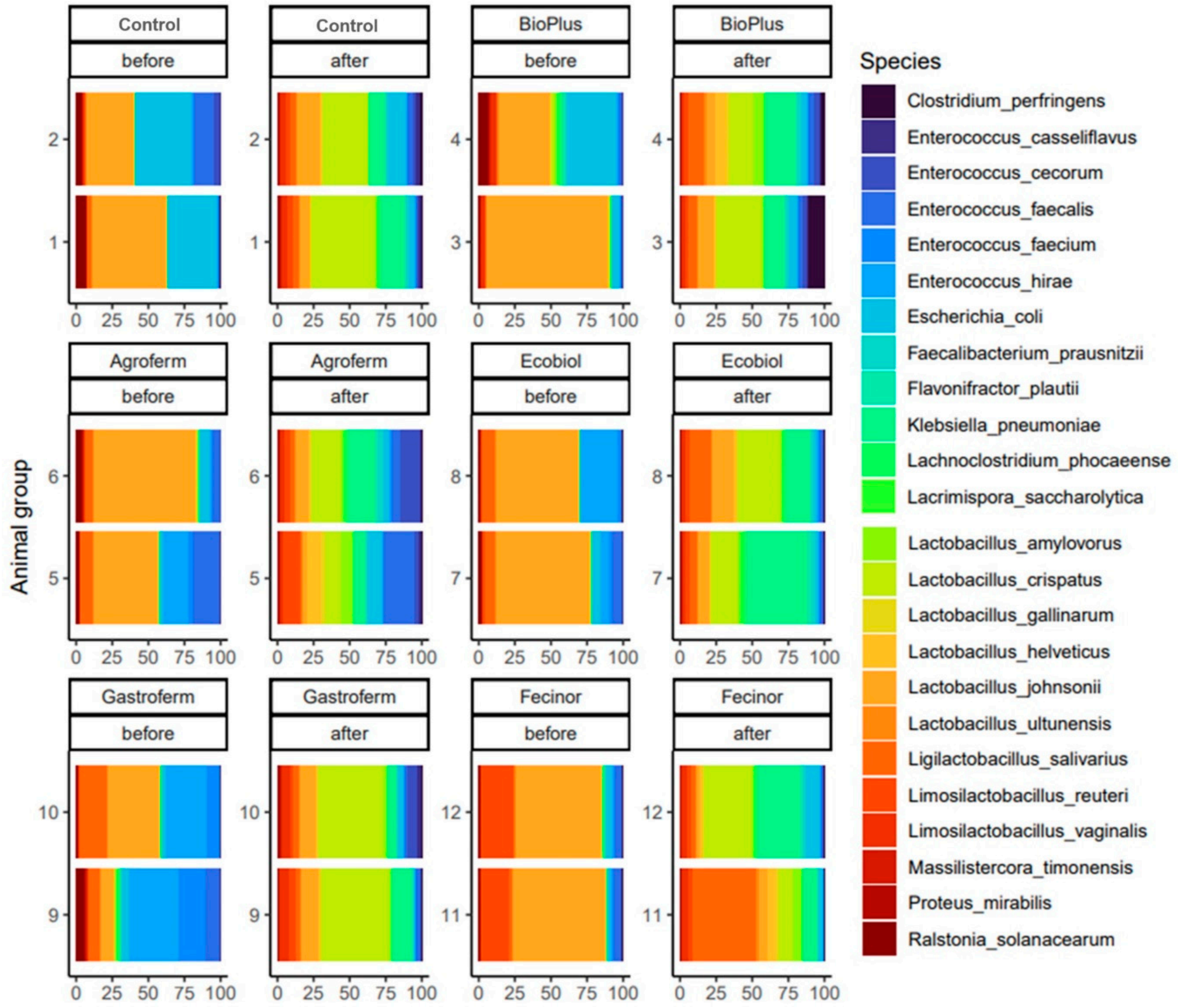

Supplementary Figure 10 Abundances of the core bacteriome per sample, defined as species present in at least 10% of the samples at a minimum abundance of 1%.

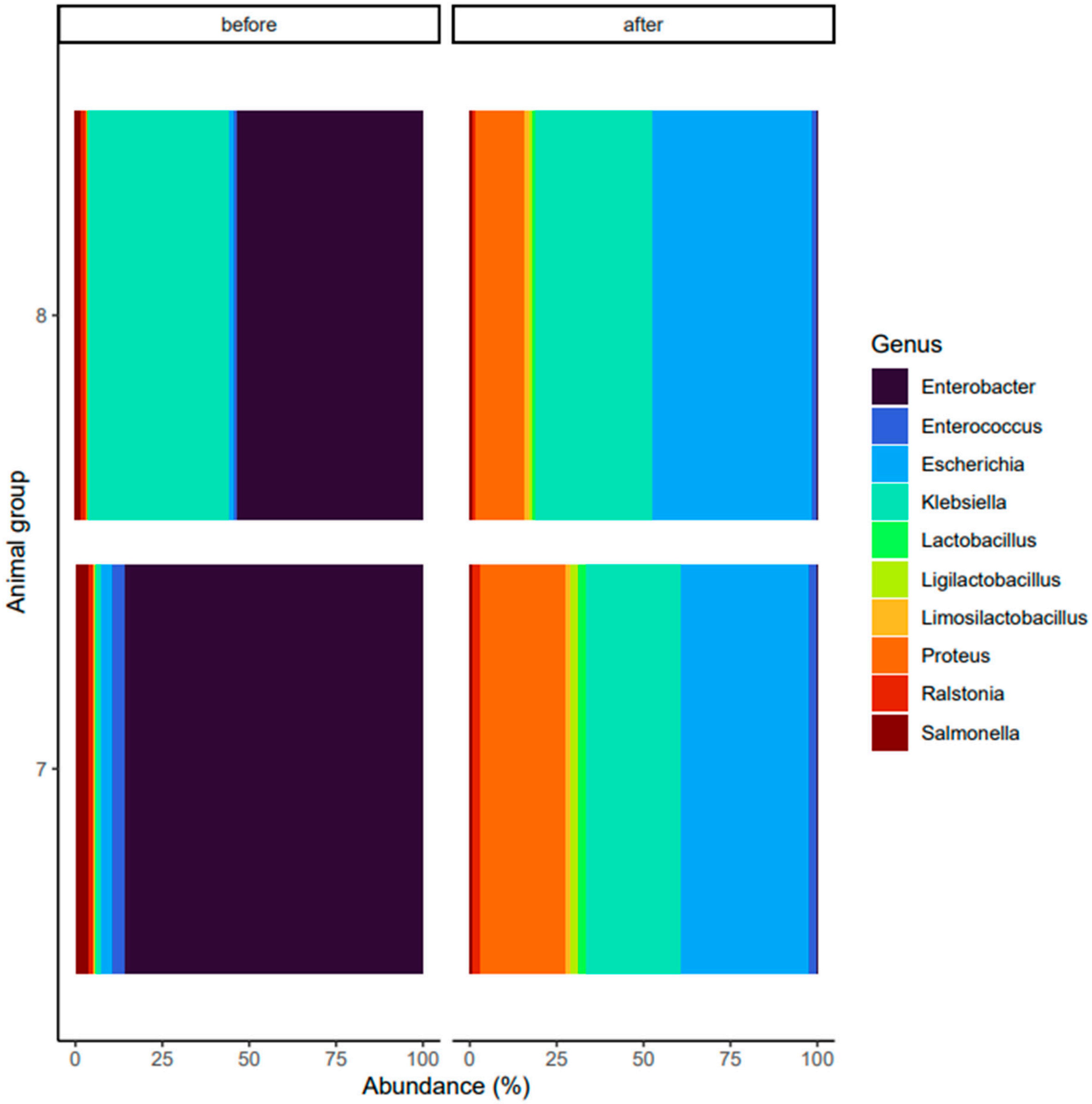

Supplementary Figure 11 Core-bacteriome abundances for enrichment samples, defined as having at least 1% of genes present in at least 10% of the samples.

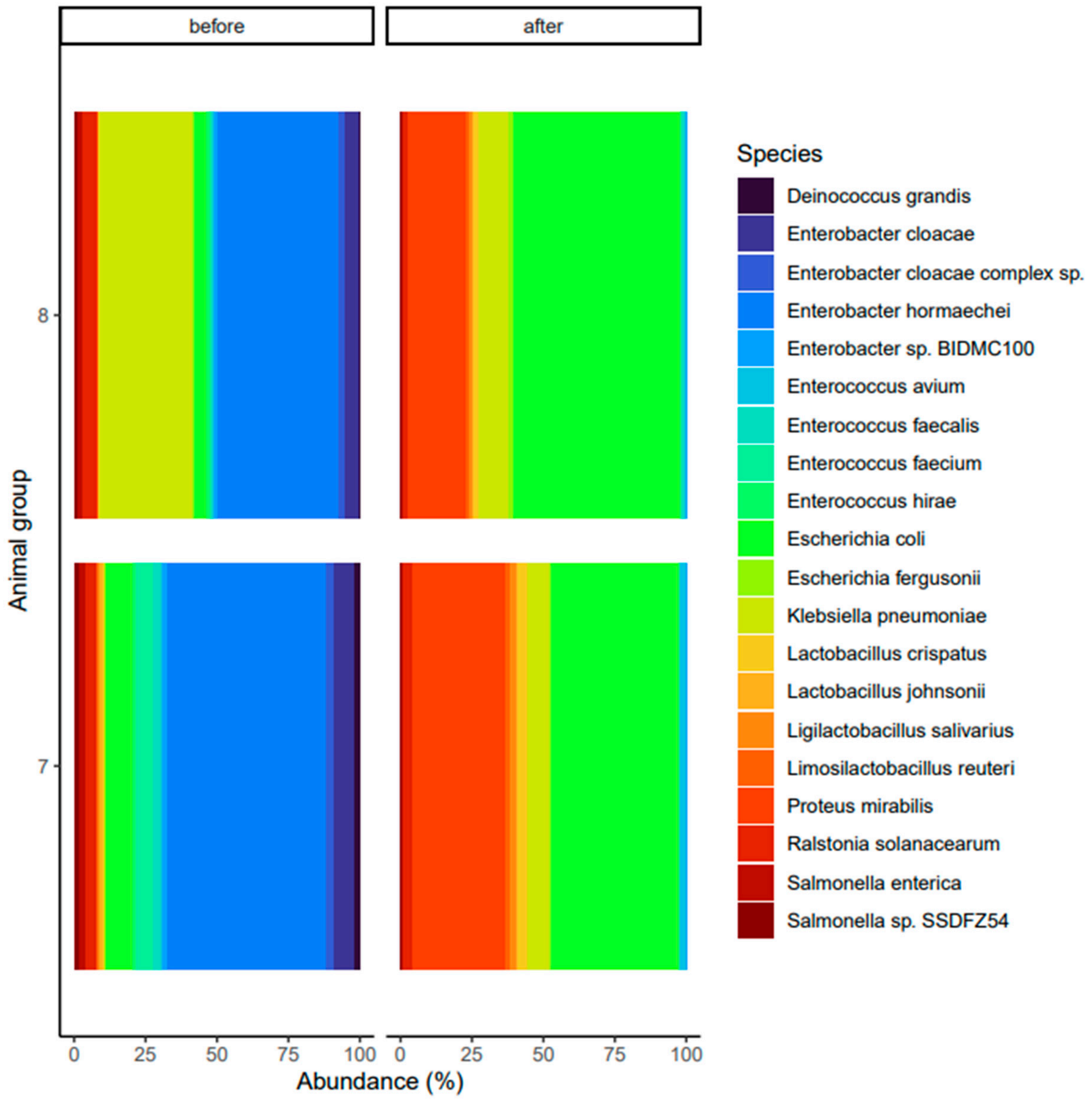

Supplementary Figure 12 Core bacteriome abundances in enrichment samples, defined by the presence of at least 1% of species in at least 10% of the samples.

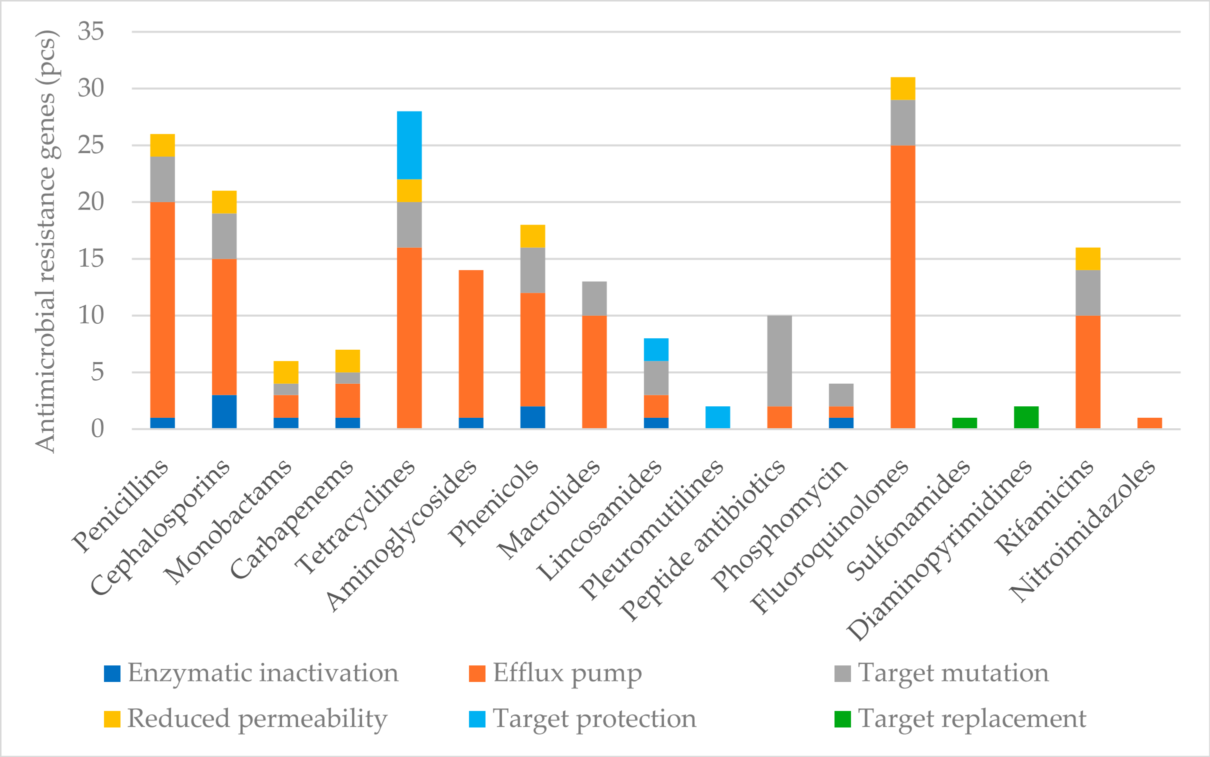

Supplementary Figure 13 Frequency of antimicrobial resistance genes (ARGs) identified during sequencing of the control group day 42 sample by drug class, based on the individual resistance mechanisms (>90% coverage).

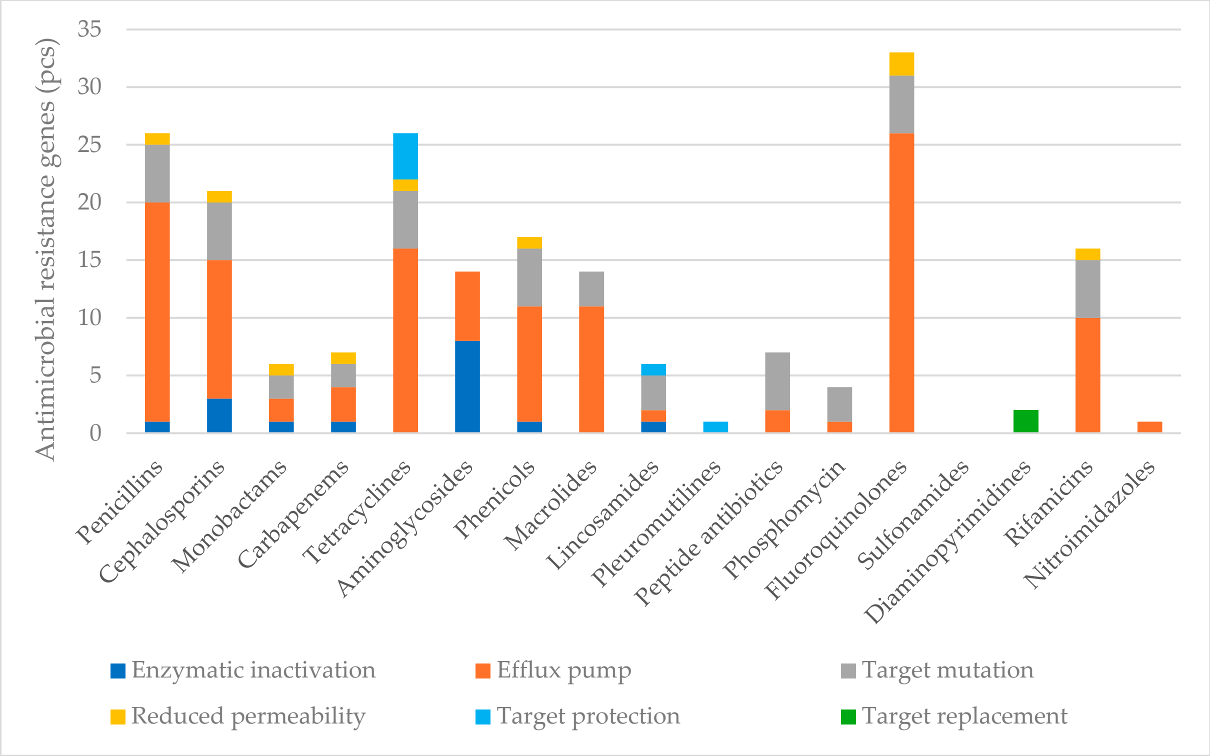

Supplementary Figure 14 Frequency of antimicrobial resistance genes (ARGs) identified during sequencing of the Bioplus group day 42 sample by drug class, based on the individual resistance mechanisms (>90% coverage).

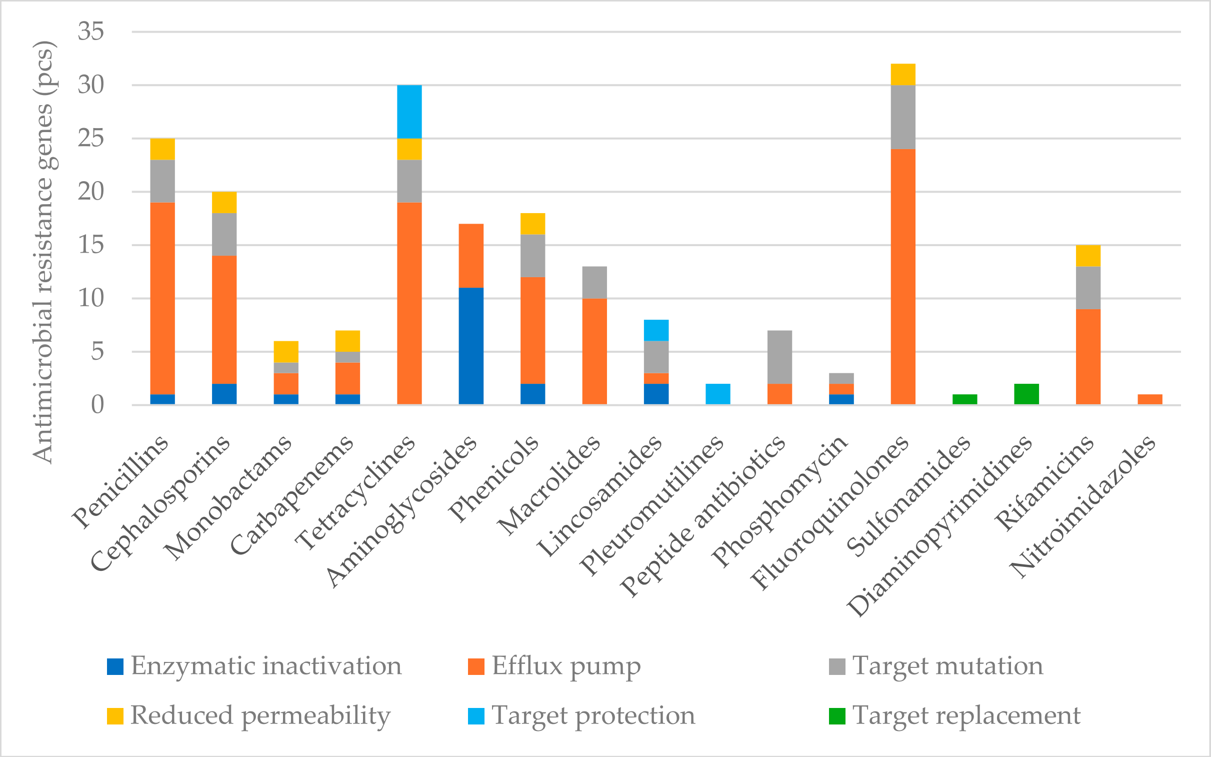

Supplementary Figure 15 Frequency of antimicrobial resistance genes (ARGs) identified during sequencing of the Agroferm group day 42 sample by drug class, based on the individual resistance mechanisms (>90% coverage).

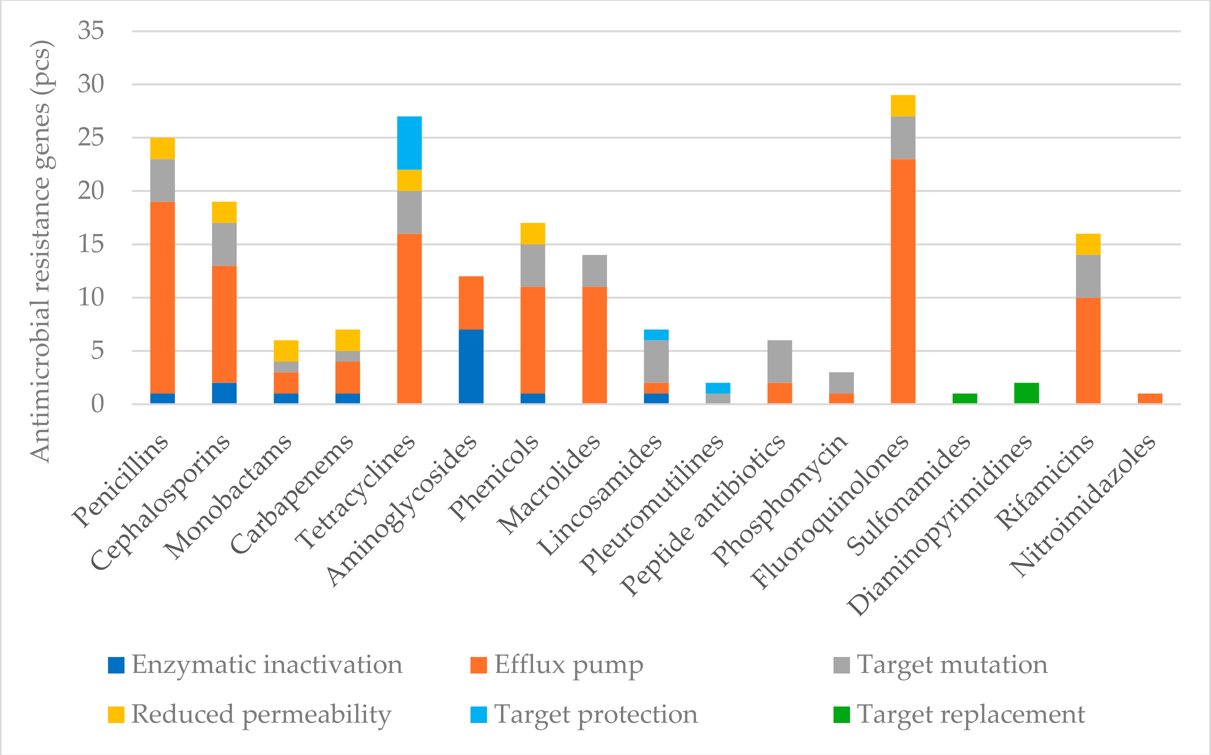

Supplementary Figure 16 Frequency of antimicrobial resistance genes (ARGs) identified during sequencing of the Ecobiol group day 42 sample by drug class, based on the individual resistance mechanisms (>90% coverage).

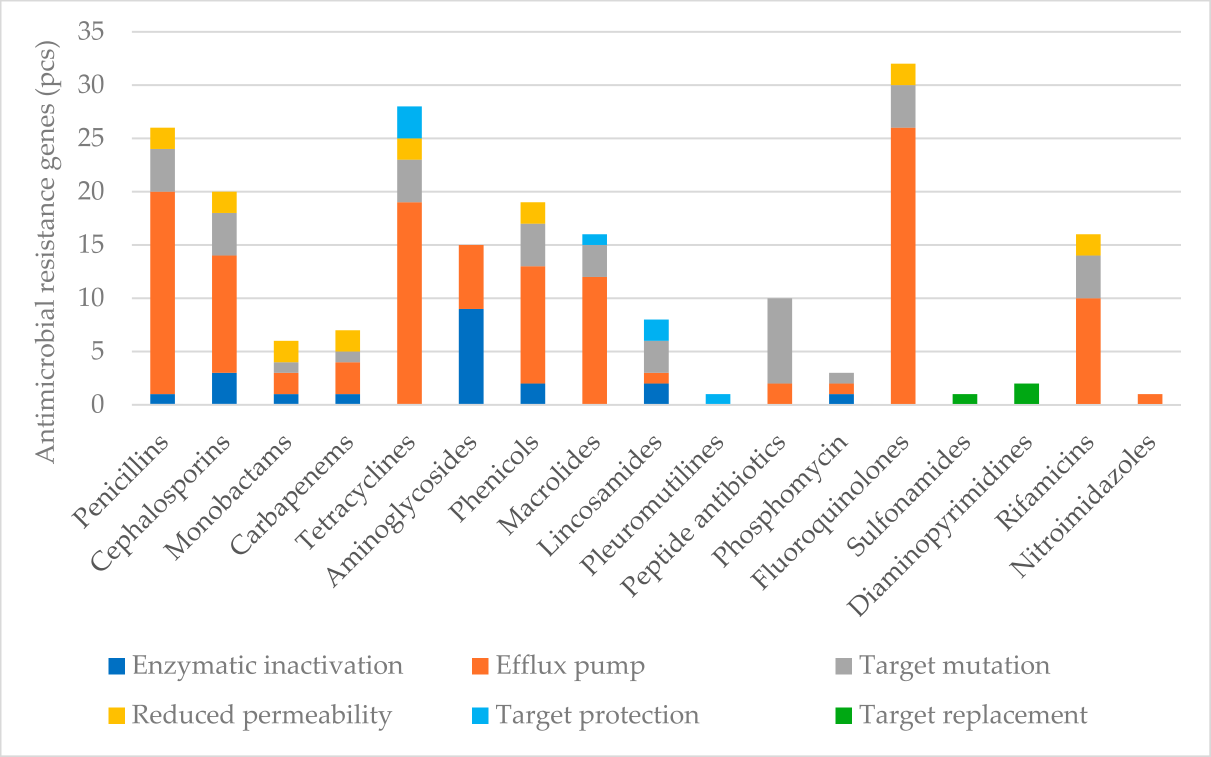

Supplementary Figure 17 Frequency of antimicrobial resistance genes (ARGs) identified during sequencing of the Gastroferm group day 42 sample by drug class, based on the individual resistance mechanisms (>90% coverage).

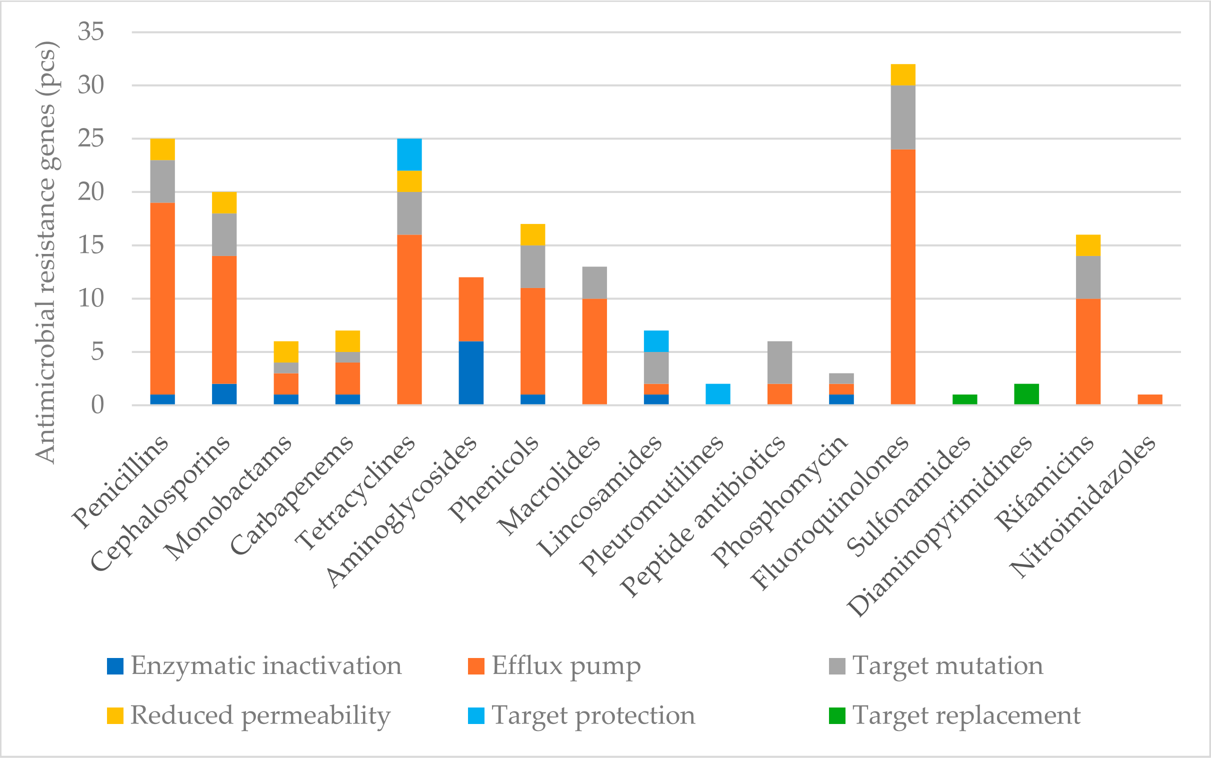

Supplementary Figure 18 Frequency of antimicrobial resistance genes (ARGs) identified during sequencing of the Fecinor group day 42 sample by drug class, based on the individual resistance mechanisms (>90% coverage).
