# Supplementary figures and images for: Comprehensive Metagenomic Analysis of Veterinary Probiotics in Broiler Chickens
Source: Animals (Basel). 2024 Jun 29;14(13):1927. doi: 10.3390/ani14131927 (PMC11240415; doi:10.3390/ani14131927)

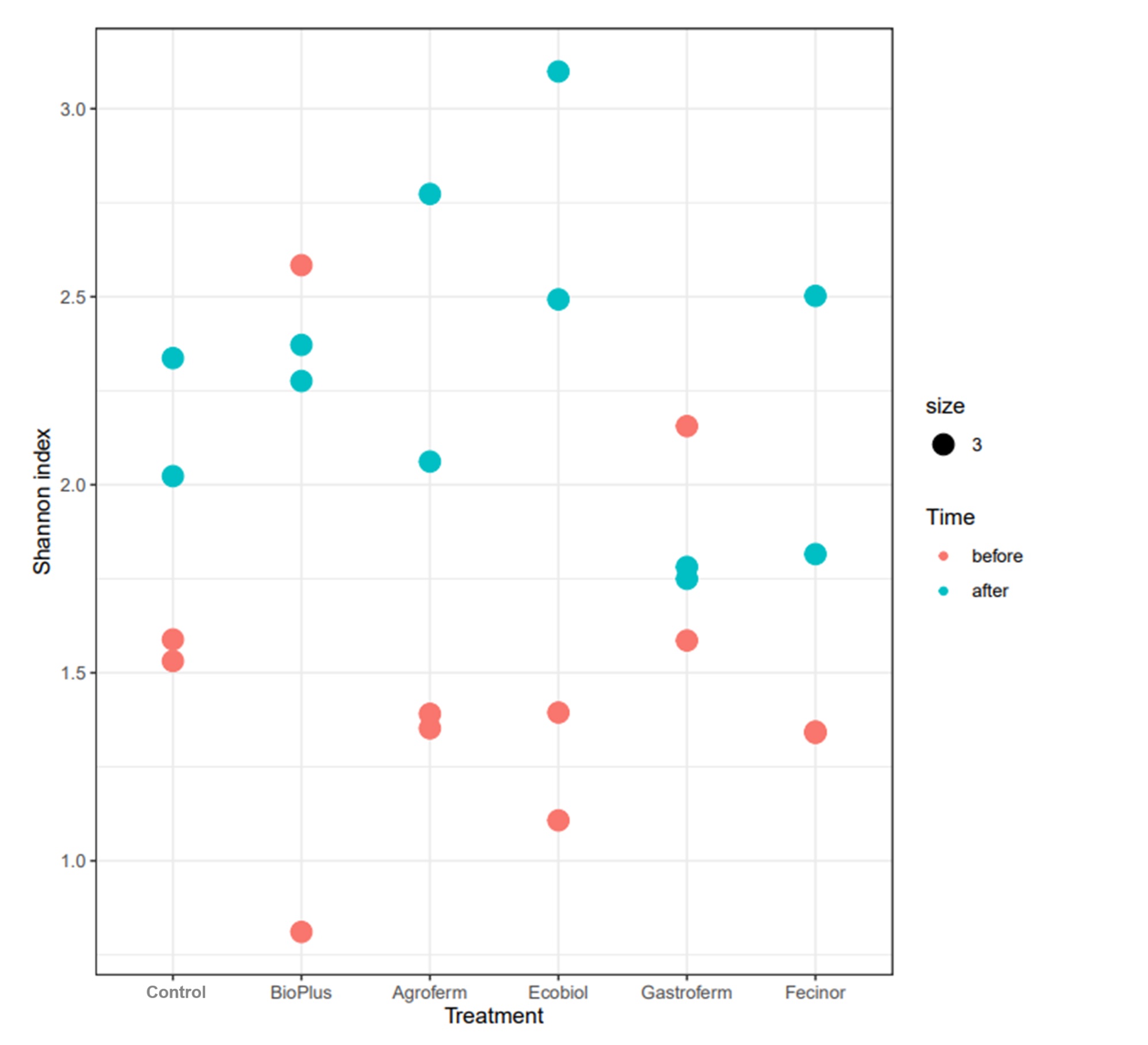

Supplement: Supplementary file 1 [file animals-14-01927-s001.zip › Supplementary Figure 1.jpg]

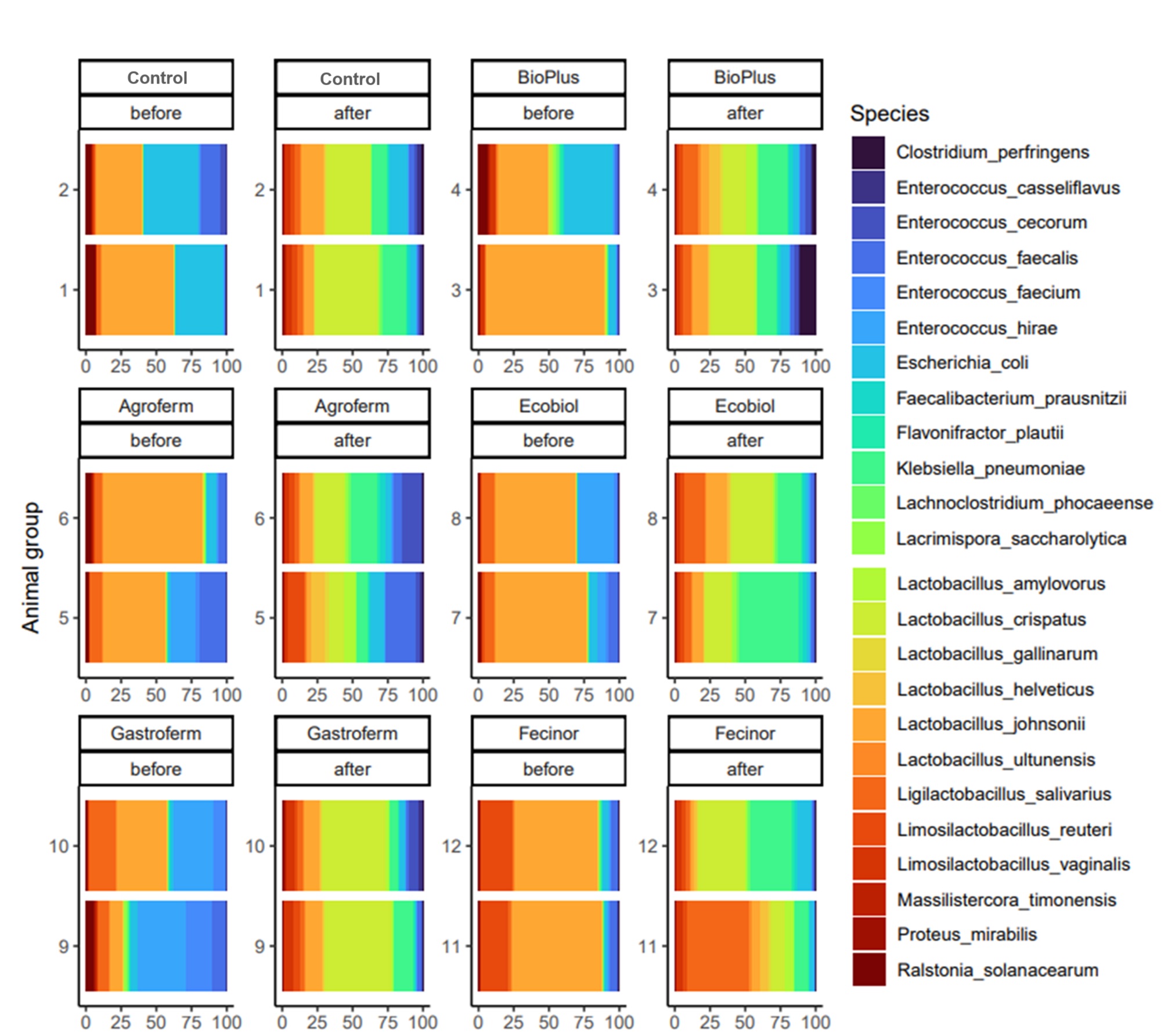

Supplement: Supplementary file 1 [file animals-14-01927-s001.zip › Supplementary Figure 10.jpg]

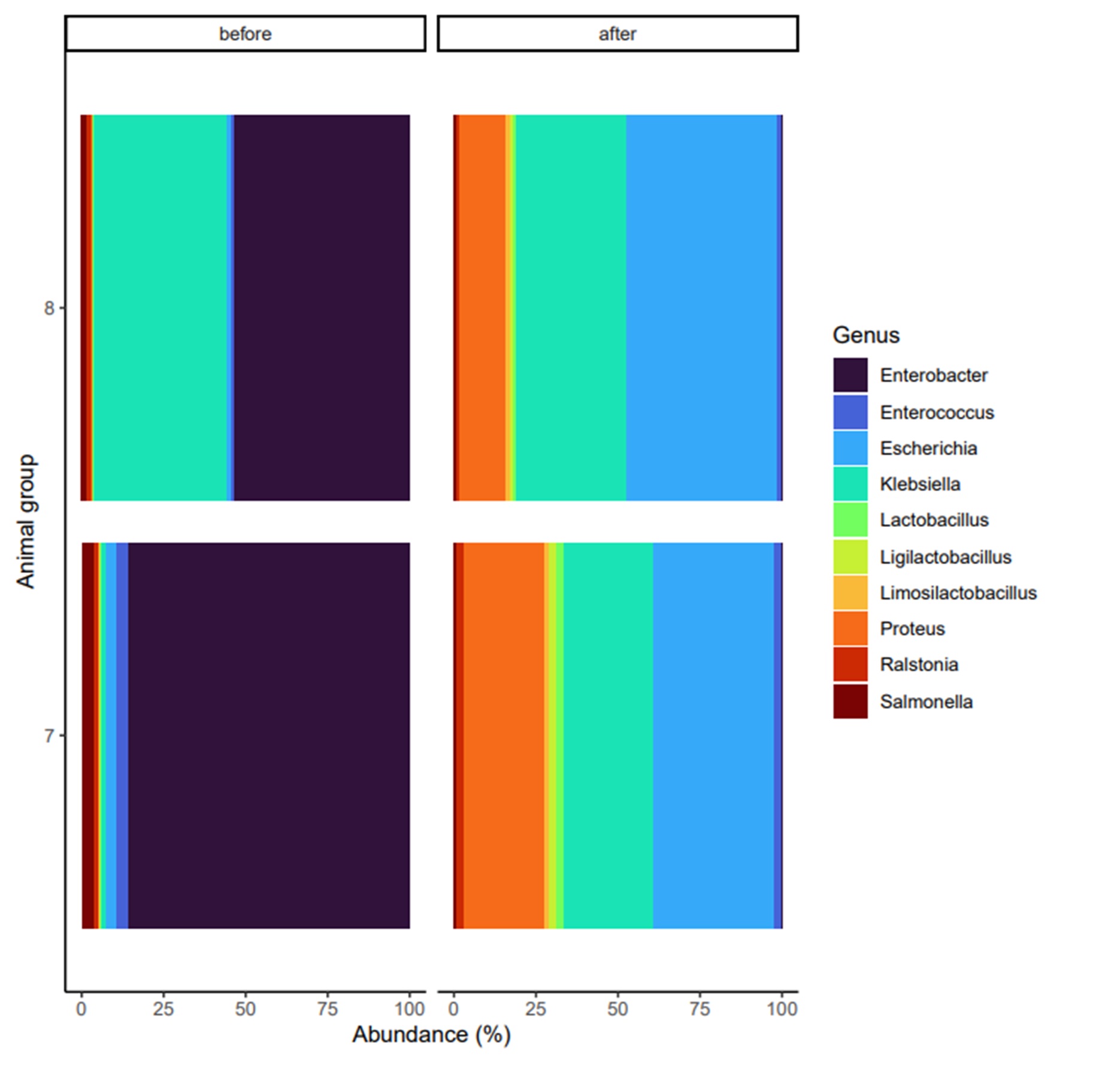

Supplement: Supplementary file 1 [file animals-14-01927-s001.zip › Supplementary Figure 11.jpg]

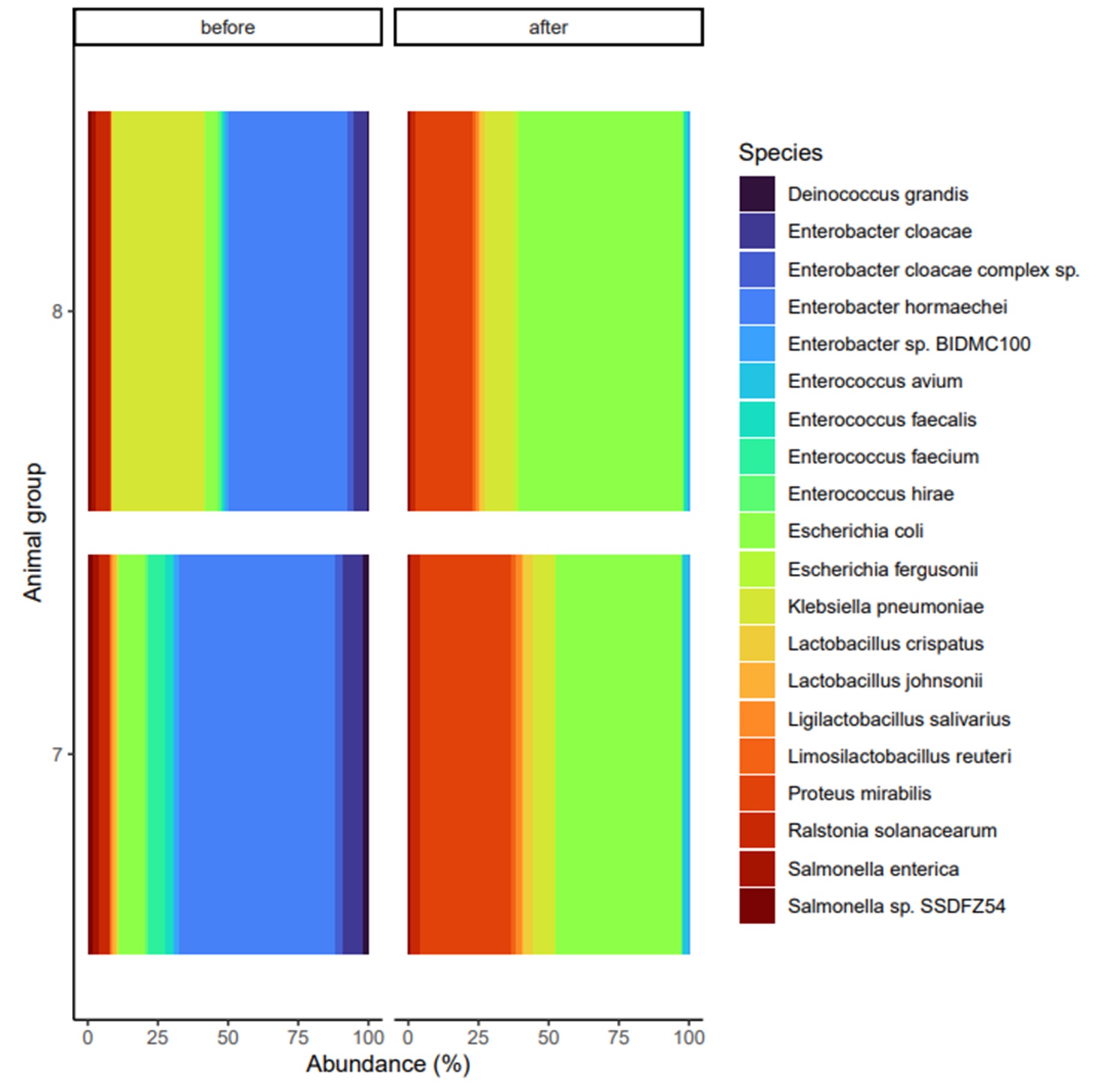

Supplement: Supplementary file 1 [file animals-14-01927-s001.zip › Supplementary Figure 12.jpg]

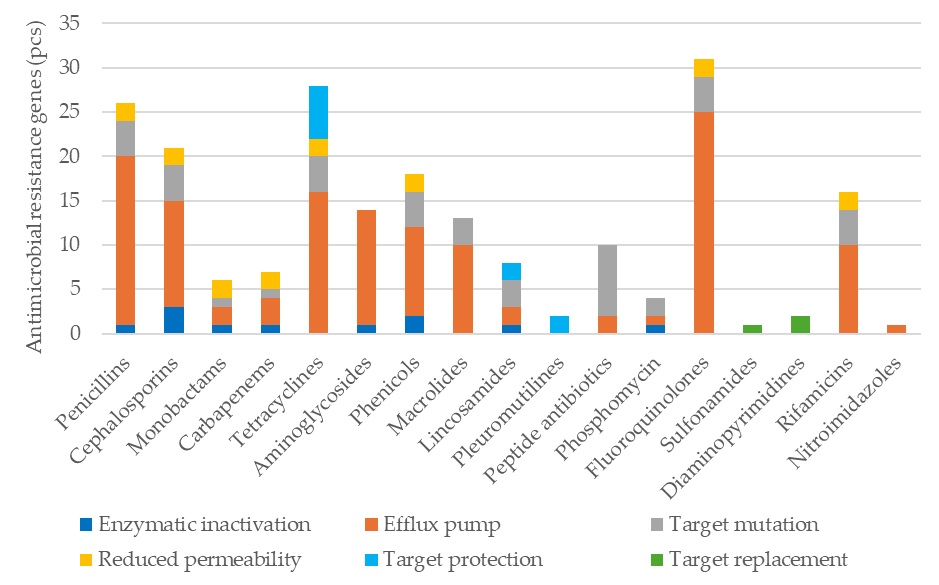

Supplement: Supplementary file 1 [file animals-14-01927-s001.zip › Supplementary Figure 13.jpg]

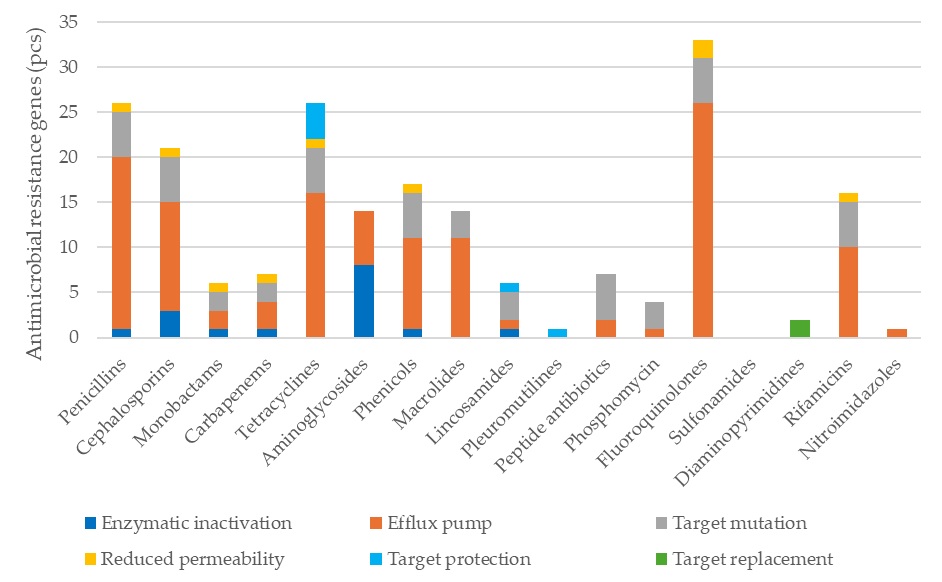

Supplement: Supplementary file 1 [file animals-14-01927-s001.zip › Supplementary Figure 14.jpg]

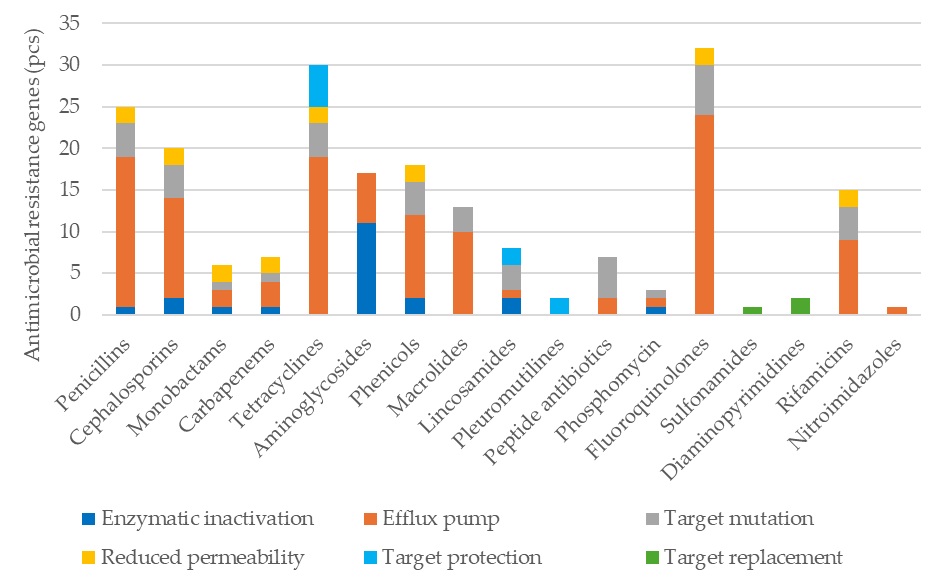

Supplement: Supplementary file 1 [file animals-14-01927-s001.zip › Supplementary Figure 15.jpg]

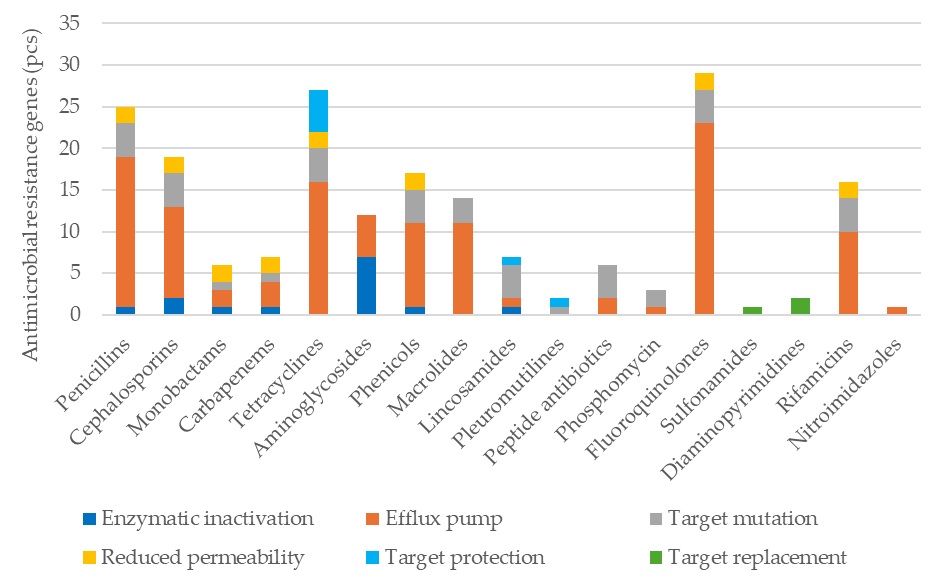

Supplement: Supplementary file 1 [file animals-14-01927-s001.zip › Supplementary Figure 16.jpg]

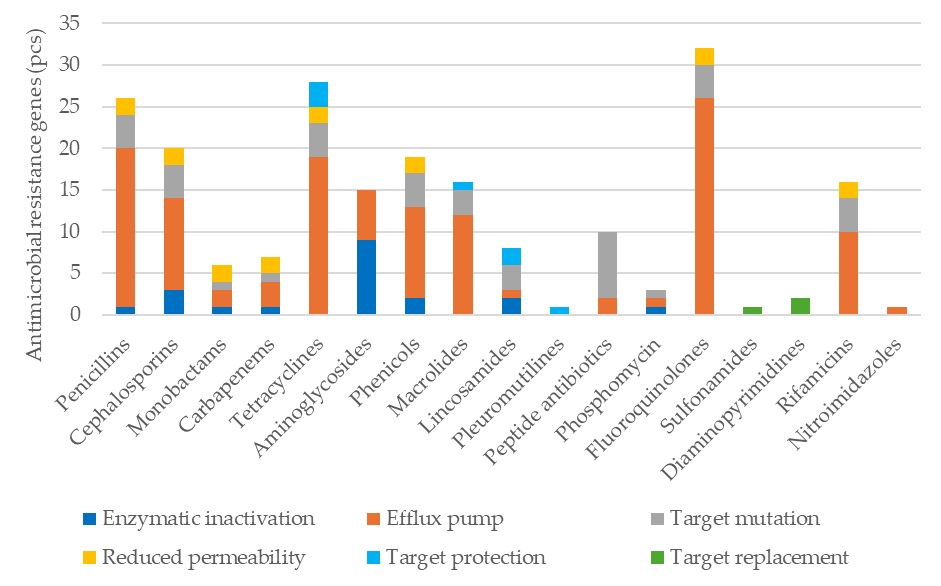

Supplement: Supplementary file 1 [file animals-14-01927-s001.zip › Supplementary Figure 17.jpg]

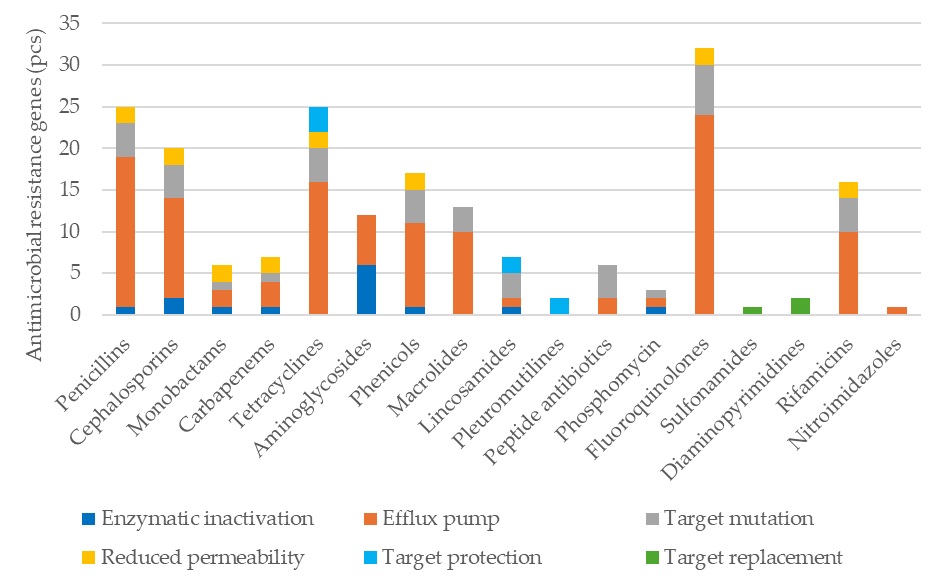

Supplement: Supplementary file 1 [file animals-14-01927-s001.zip › Supplementary Figure 18.jpg]

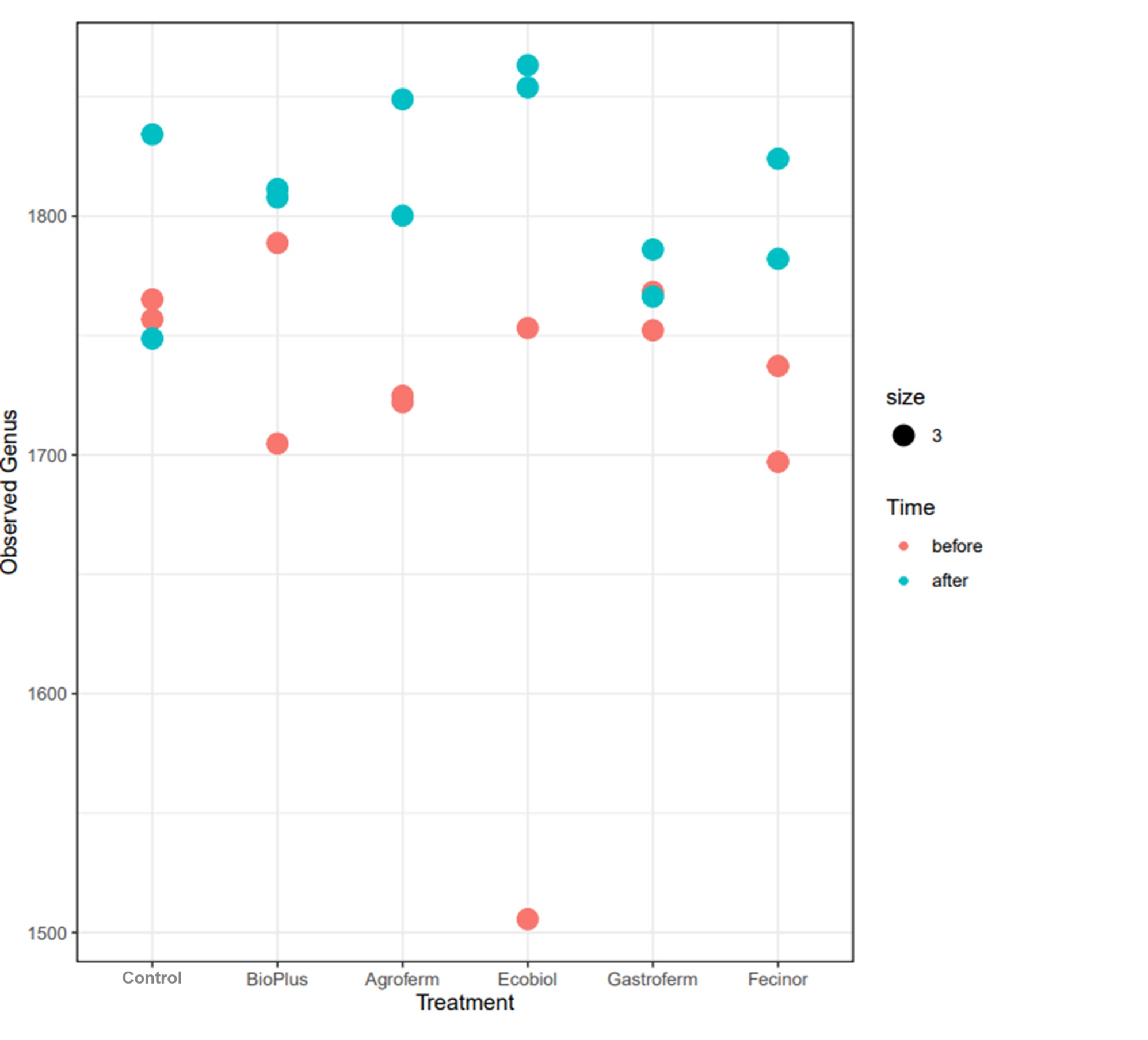

Supplement: Supplementary file 1 [file animals-14-01927-s001.zip › Supplementary Figure 2.jpg]

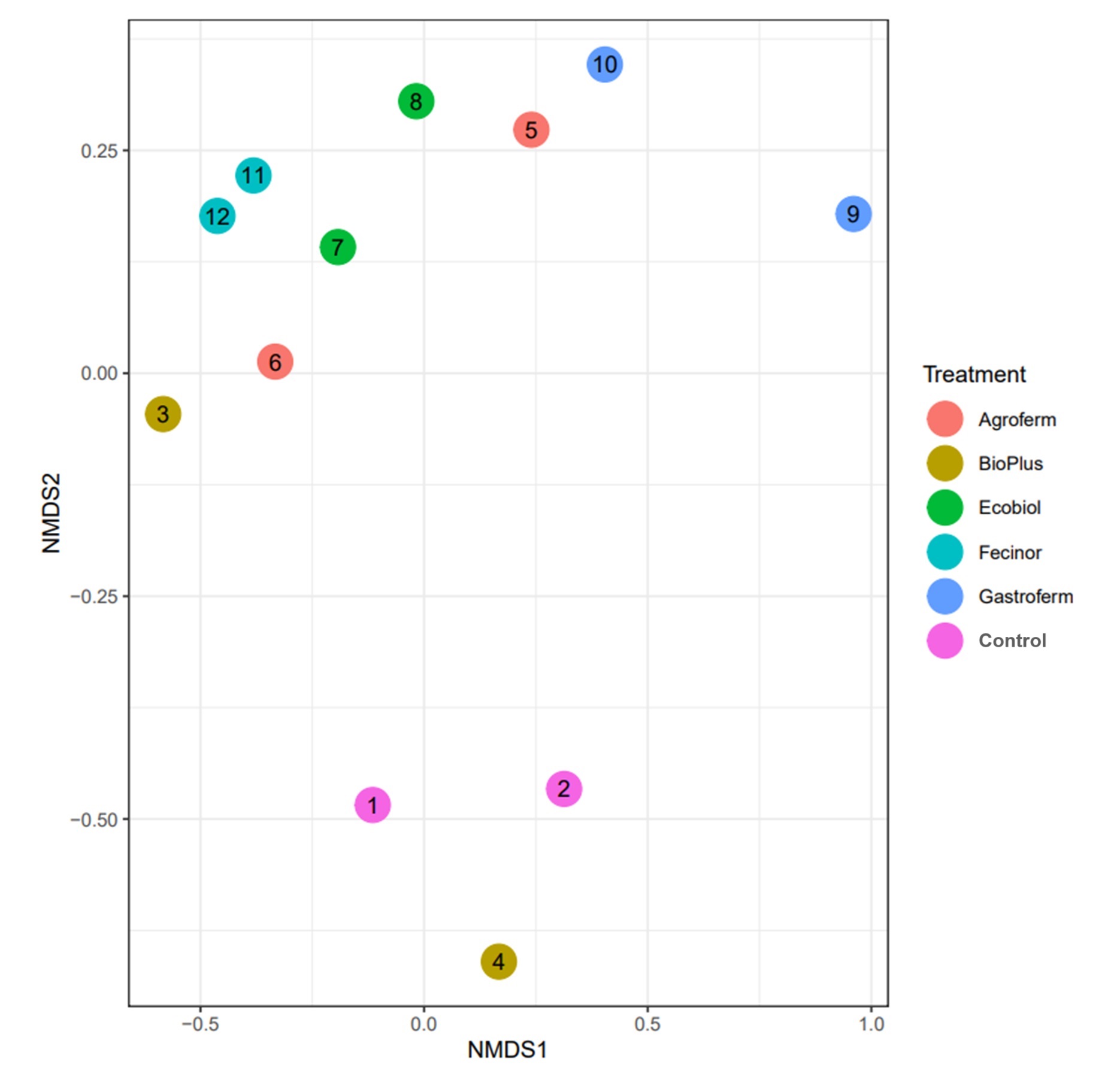

Supplement: Supplementary file 1 [file animals-14-01927-s001.zip › Supplementary Figure 3.jpg]

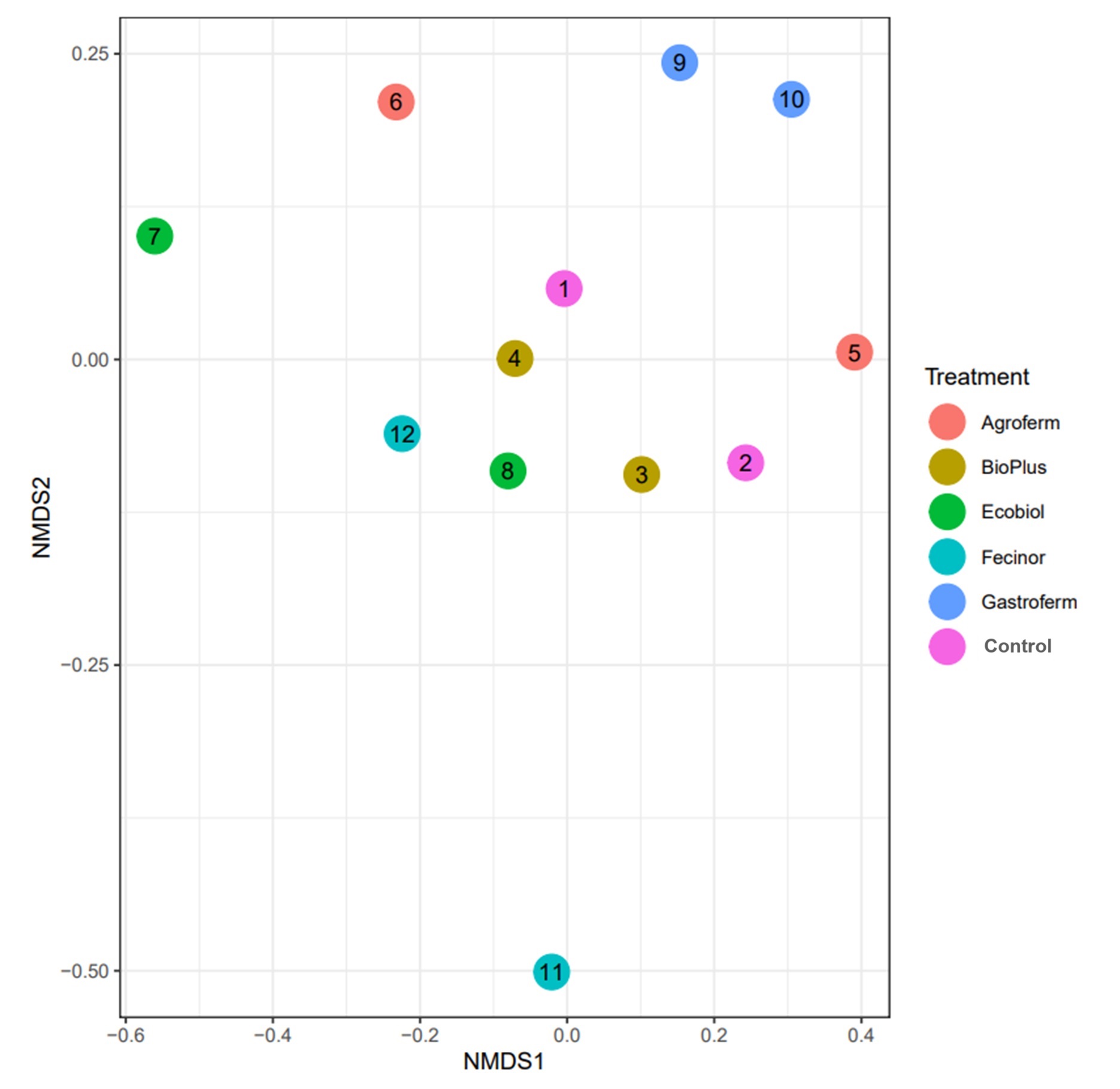

Supplement: Supplementary file 1 [file animals-14-01927-s001.zip › Supplementary Figure 4.jpg]

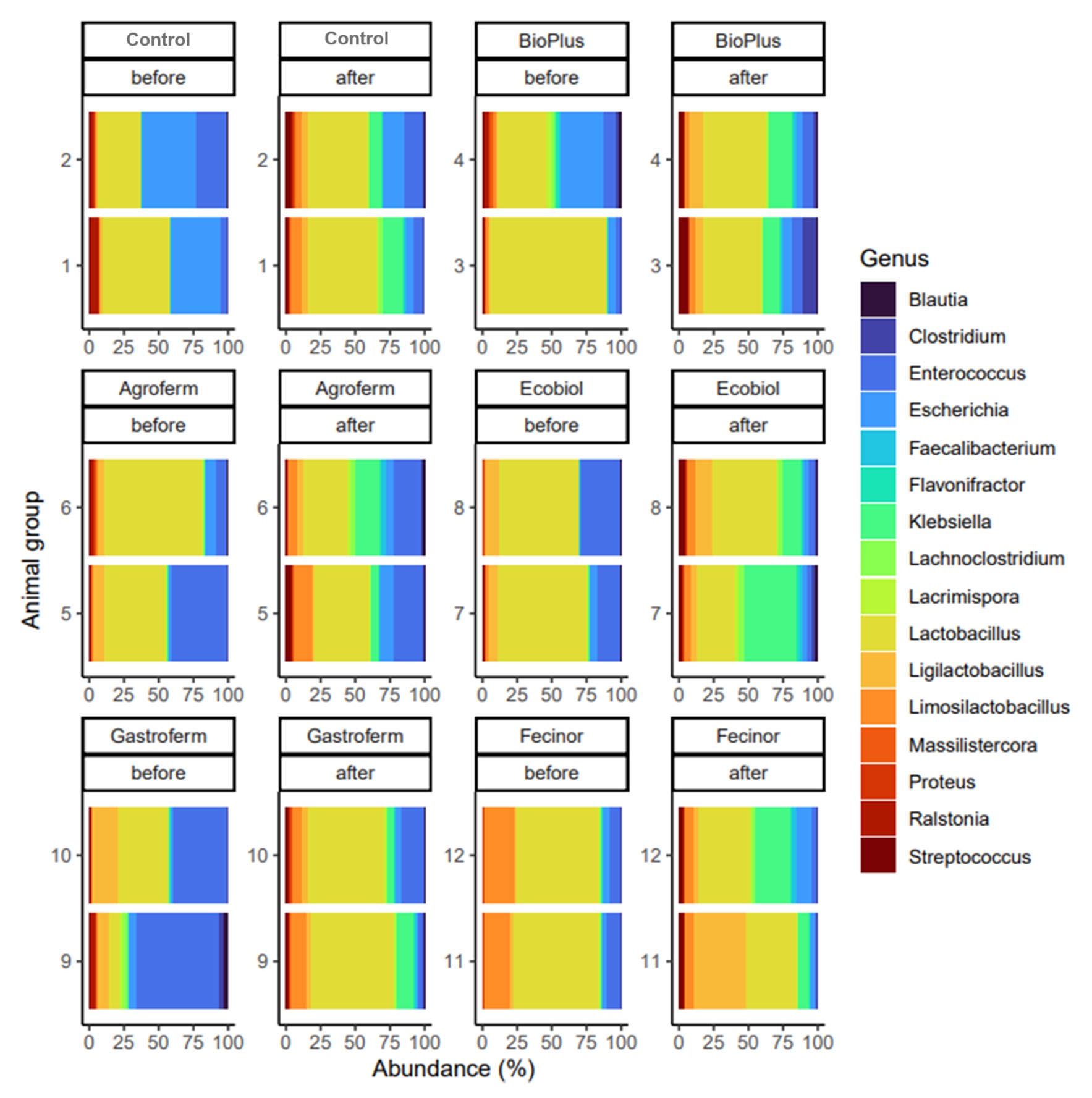

Supplement: Supplementary file 1 [file animals-14-01927-s001.zip › Supplementary Figure 5.jpg]

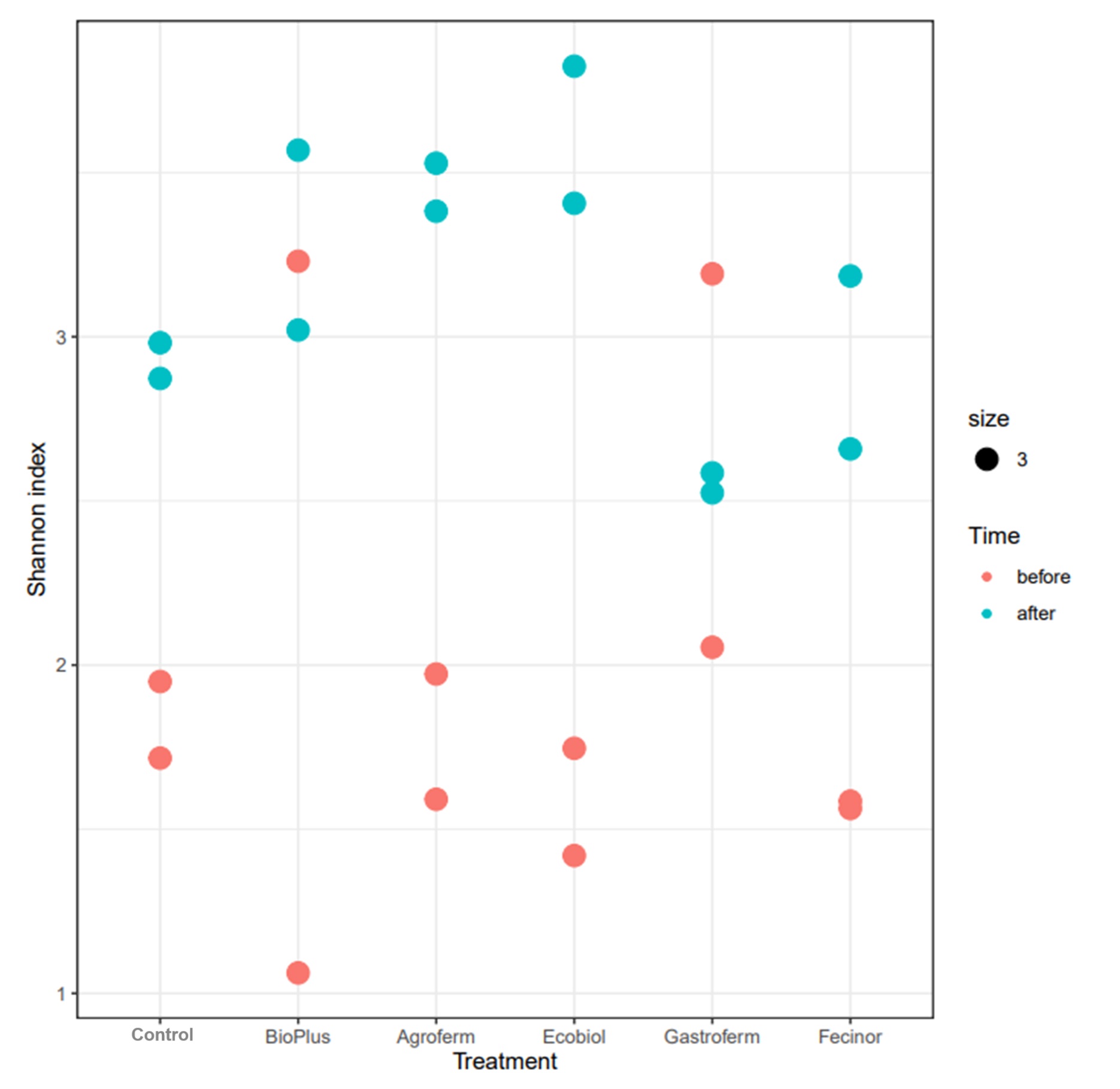

Supplement: Supplementary file 1 [file animals-14-01927-s001.zip › Supplementary Figure 6.jpg]

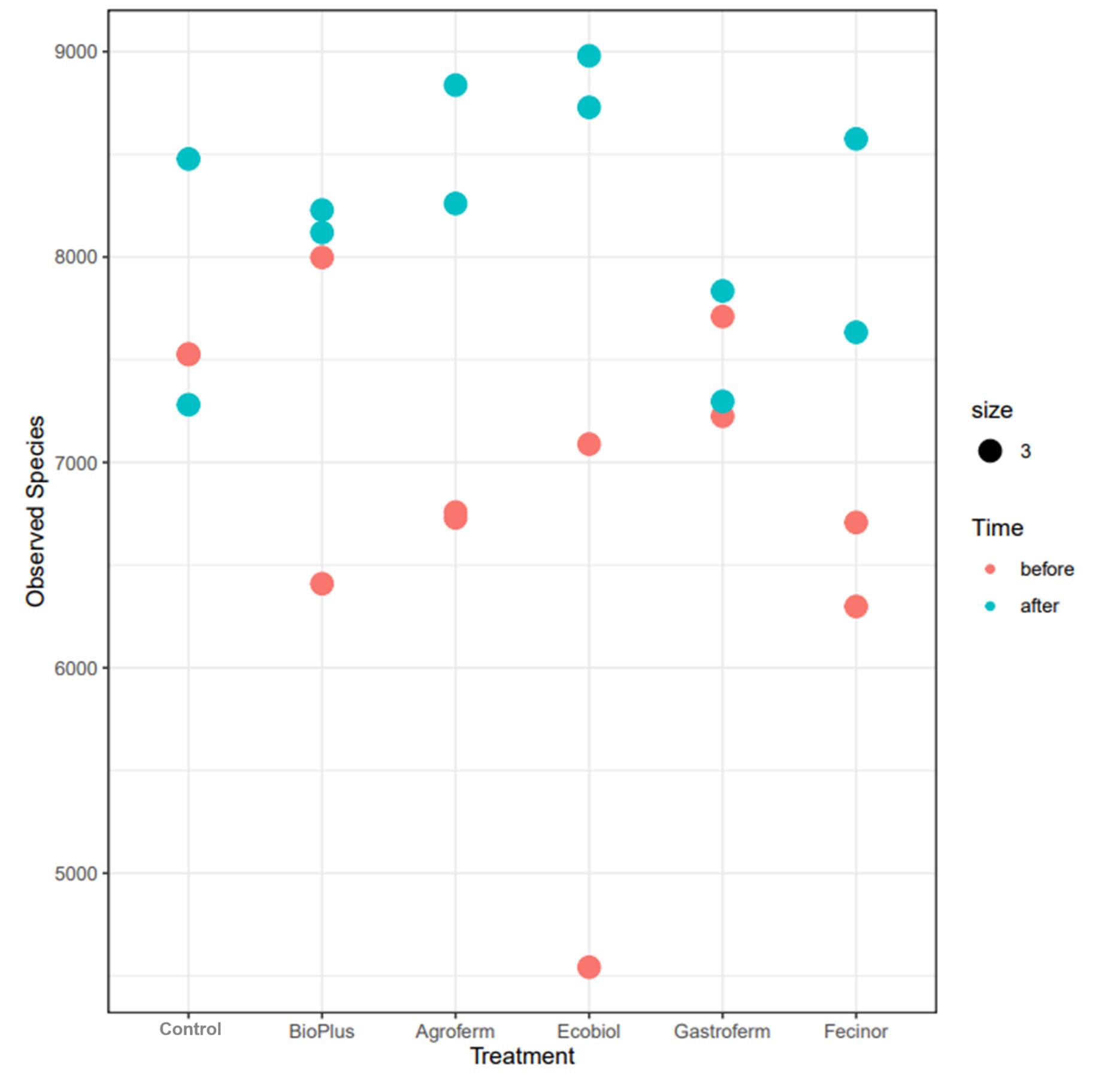

Supplement: Supplementary file 1 [file animals-14-01927-s001.zip › Supplementary Figure 7.jpg]

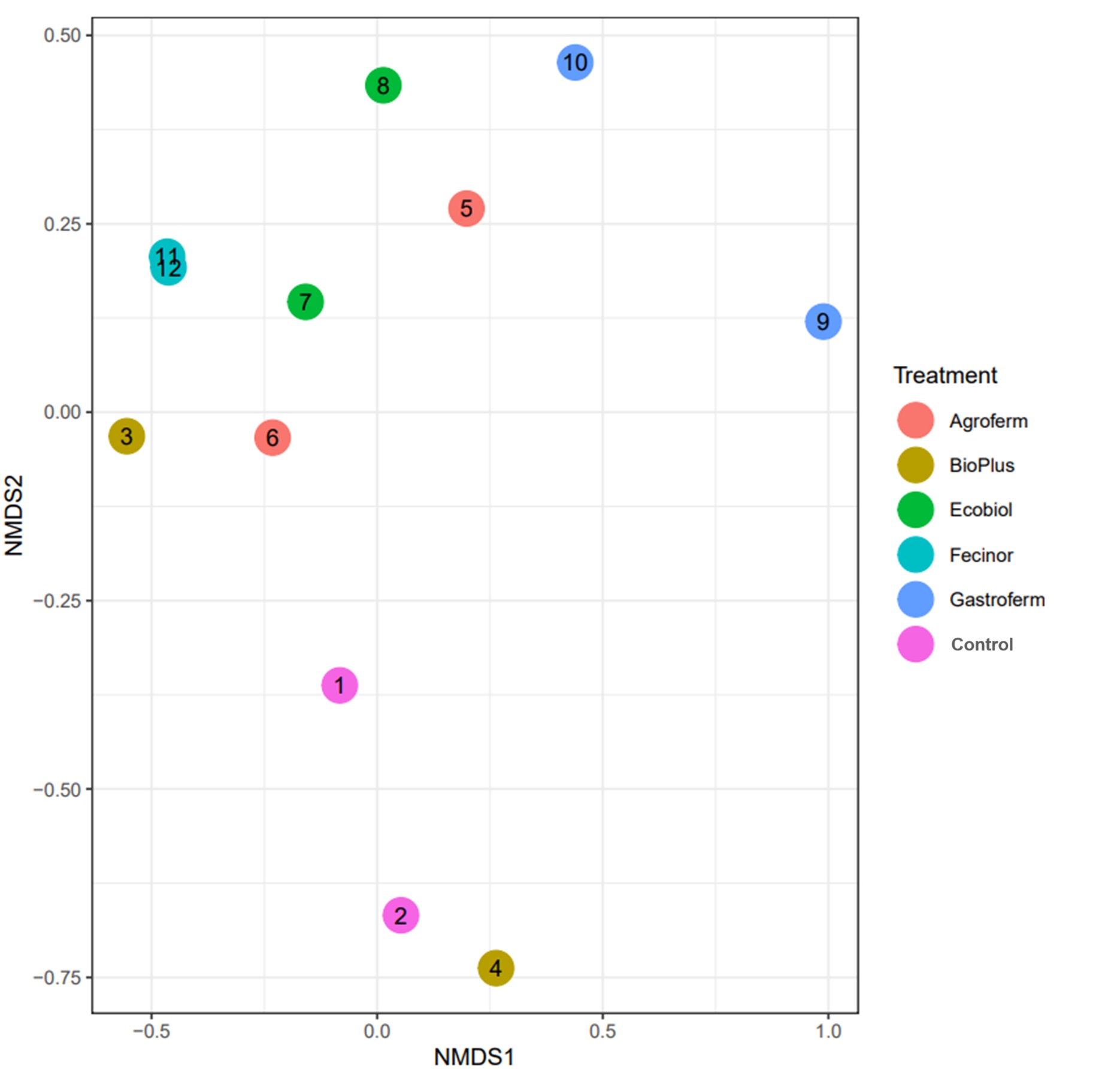

Supplement: Supplementary file 1 [file animals-14-01927-s001.zip › Supplementary Figure 8.jpg]

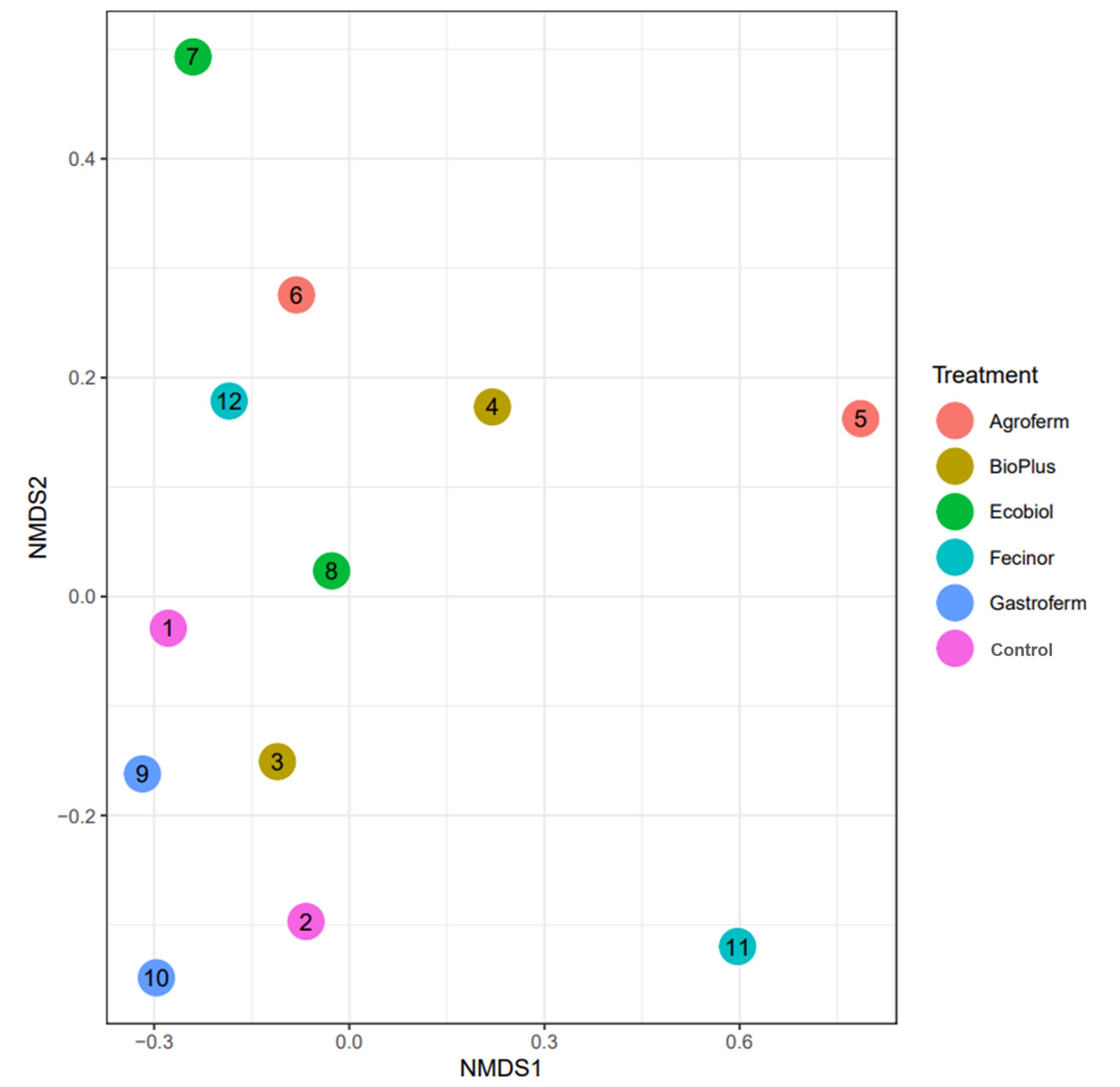

Supplement: Supplementary file 1 [file animals-14-01927-s001.zip › Supplementary Figure 9.jpg]
